# The stress sigma factor σ^S^/RpoS counteracts Fur repression of genes involved in iron and manganese metabolism and modulates the ionome of *Salmonella enterica* serovar Typhimurium

**DOI:** 10.1371/journal.pone.0265511

**Published:** 2022-03-31

**Authors:** Selma Metaane, Véronique Monteil, Sophie Ayrault, Louise Bordier, Corinne Levi-Meyreuis, Françoise Norel

**Affiliations:** 1 Institut Pasteur, Université de Paris, CNRS UMR3528, Biochimie des Interactions Macromoléculaires, F-75015, Paris, France; 2 Laboratoire des Sciences du Climat et de l’Environnement, LSCE/IPSL, CEA-CNRS-UVSQ, Université Paris-Saclay, 91191, Gif-sur-Yvette, France; 3 Université Paris-Saclay, 91400, Orsay, France; East Carolina University Brody School of Medicine, UNITED STATES

## Abstract

In many Gram-negative bacteria, the stress sigma factor of RNA polymerase, σ^S^/RpoS, remodels global gene expression to reshape the physiology of quiescent cells and ensure their survival under non-optimal growth conditions. In the foodborne pathogen *Salmonella enterica* serovar Typhimurium, σ^S^ is also required for biofilm formation and virulence. We have previously identified sRNAs genes positively controlled by σ^S^ in *Salmonella*, including the two paralogous sRNA genes, *ryhB1* and *ryhB2*/*isrE*. Expression of *ryhB1* and *ryhB2* is repressed by the ferric uptake regulator Fur when iron is available. In this study, we show that σ^S^ alleviates Fur-mediated repression of the *ryhB* genes and of additional Fur target genes. Moreover, σ^S^ induces transcription of the manganese transporter genes *mntH* and *sitABCD* and prevents their repression, not only by Fur, but also by the manganese-responsive regulator MntR. These findings prompted us to evaluate the impact of a Δ*rpoS* mutation on the *Salmonella* ionome. Inductively coupled plasma mass spectrometry analyses revealed a significant effect of the Δ*rpoS* mutation on the cellular concentration of manganese, magnesium, cobalt and potassium. In addition, transcriptional fusions in several genes involved in the transport of these ions were regulated by σ^S^. This study suggests that σ^S^ controls fluxes of ions that might be important for the fitness of quiescent cells. Consistent with this hypothesis, the Δ*rpoS* mutation extended the lag phase of *Salmonella* grown in rich medium supplemented with the metal ion chelator EDTA, and this effect was abolished when magnesium, but not manganese or iron, was added back. These findings unravel the importance of σ^S^ and magnesium in the regrowth potential of quiescent cells.

## Introduction

In many Gram-negative bacteria, the alternative sigma subunit of RNA polymerase, σ^S^/RpoS, remodels global gene expression to reshape the cell physiology and ensure survival under starvation and various stress conditions (the so-called general stress response) [1]. The σ^S^ network has been intensively studied in the model organism, *Escherichia coli* K-12 (*E*. *coli*) [1–3]. In the closely related foodborne pathogen *Salmonella enterica* serovar Typhimurium (*S*. Typhimurium), σ^S^ is required for stress resistance, biofilm formation and virulence [1,4]. Global transcriptomic studies have revealed the composition of the *Salmonella* σ^S^ network, and a major effect of σ^S^ on remodeling of membrane and metabolic functions, and have highlighted the importance of down-regulation of gene expression by σ^S^ [5,6].

Until recently, the σ^S^ response was believed to be predominantly transcriptional. However, using a MS-based proteomics approach to unravel the impact of σ^S^ on global protein production, we identified a large number of genes down-regulated at the protein level, but not at the transcript level, suggesting that post-transcriptional regulation plays a larger role in σ^S^ gene regulation than previously recognized [7]. Small RNAs (sRNAs) might be mediators in some of these post-transcriptional effects. Indeed, we have identified several sRNAs genes under positive control by σ^S^ in ATCC14028 [5]. σ^S^-dependent expression of most of these sRNAs has been subsequently observed by Colgan *et al*. in another strain of *S*. Typhimurium [8]. Analysis of gene expression in 18 mutants in regulatory genes, including *rpoS*, was reported in the *Salmonella* Gene Expression Compendium (SalCom) database [8], and we noticed that several σ^S^-activated sRNA genes were up-regulated in a Δ*fur* mutant (S1 Fig) deficient for the iron-binding global ferric uptake regulator Fur. This observation prompted us to address the possibility that σ^S^ alleviates Fur-mediated repression of those sRNAs genes when iron is available. The present study addresses this hypothesis focusing on two paralogous sRNA genes, *ryhB1* and *ryhB2/isrE*.

RyhB homologs are found in several bacterial genera [9]. In *E*. *coli*, the primary function of RyhB is in the iron-sparing response [9–11]. Under iron-rich conditions, RyhB expression is repressed by Fur [12,13]. Upon iron starvation, RyhB is produced and prevents the expression of nonessential iron-using proteins to allow a better usage of the newly acquired iron by essential proteins [9,10]. *S*. Typhimurium carries two *ryhB* orthologs; *ryhB1* (96 bps) that is surrounded by the same flanking genes as in *E*. *coli*, and *ryhB2* (*isrE*, 98 bps) that is located on a pathogenicity island [14]. There is a 33 bp sequence of perfect homology between RyhB1 and RyhB2 [14]. The production of both sRNAs is influenced in response to iron-availability by the activity of Fur and is induced by σ^S^ during late stationary phase in rich medium [5,8,14]. The *Salmonella* RyhB sRNAs are induced upon invasion of host cells and appear to be involved in diverse functions including acid resistance, oxidative and nitrosative stress resistance, motility and intracellular survival [12,14–19].

In this study, we show that σ^S^ counteracts Fur-mediated repression of the *Salmonella ryhB* genes and of additional Fur targets. Moreover, σ^S^ alleviates repression of manganese transporter genes, not only by Fur, but also by the manganese transport regulator MntR. Consistent with a global role for σ^S^ in ion trafficking, analysis of the *Salmonella* ionome by inductively coupled plasma mass spectrometry demonstrated that the cell content in manganese, magnesium, cobalt and potassium is affected by the Δ*rpoS* mutation. In addition, transcriptional fusions in genes involved in the transport of these ions through the inner membrane were regulated by σ^S^. These findings suggest that σ^S^ controls the homeostasis of metal ions modulating the survival and/or regrowth potential of quiescent cells. In agreement with this hypothesis, σ^S^ was required for optimal regrowth of quiescent cells in rich medium in the presence of the metal ion chelating agent EDTA, and our data pinpoint to a major role for magnesium in this phenomenon.

## Material and methods

### Bacterial strains, bacteriophage, plasmids and growth conditions

Strains and plasmids are listed in S1 Table. Bacteriophage P22HT105/1*int* was used to transfer mutations and *lacZ* fusions between *Salmonella* strains by transduction [20]. Green plates, for screening for P22-infected cells or lysogens, were prepared as described previously [21]. Bacteria were routinely grown in LB medium [22] at 37°C under aeration. When indicated, the LB medium was supplemented with the iron chelator 2,2′-dipyridyl (DP), the metal chelating agent ethylenediaminetetraacetic acid (EDTA), magnesium chloride (MgCl_2_), iron chloride (FeCl_2_) and manganese chloride (MnCl_2_), at the indicated concentrations. Antibiotics were used at the following concentrations (in *μ*g per ml): carbenicillin (Cb), 100; chloramphenicol, (Cm) 15 for the chromosomal resistance gene and 30 for the plasmid resistance gene; kanamycin, (Km) 50; and tetracycline, (Tc) 20.

### DNA manipulations, *lacZ* fusions and inactivation of chromosomal genes

Standard molecular biology techniques were used [22,23]. Oligonucleotides were obtained from Sigma-Aldrich and are listed in S2 Table. Functional annotations and DNA sequences of ATCC14028 genes were obtained from the KEGG server (www.genome.jp/kegg/kegg2.html). DNA sequencing was performed by Eurofins Genomics (Cologne, Germany). Chromosomal deletions and *lacZ* fusions were generated in *Salmonella* ATCC14028 using PCR-generated linear DNA fragments (S2 Table) and λ-Red recombination-based method [5,6,24–26]. All strains were confirmed to contain the expected mutation by DNA sequencing.

### Enzymatic assays

β-galactosidase activity was measured as described by Miller [27] and is expressed in Miller units which normalizes the enzymatic activity to the culture OD_600_.

### Northern analysis

Total RNA was isolated from *Salmonella* strains grown aerobically until late stationary phase (18h growth) in LB at 37°C, using TRIzol as previously described [5]. Total RNA was fractionated on an 8% polyacrylamide–7M urea gel and transferred to Hybond-N+ membranes (RPN1520B GE Healthcare). Blots were hybridized to DNA oligonucleotides specific to the RyhB1, RyhB2 and 5S sRNAs (S2 Table) labeled at the 5′ ends with T4 polynucleotide kinase using the UltraHyb-OLIGO buffer (AM8663, Ambion). ImageJ (http://rsb.info.nih.gov/ij/index.html) was used to compare the density of bands.

### Inductively coupled plasma mass spectrometry (ICP-MS)

Cell-associated contents of twenty-three cell-associated elements were measured as follows. To minimize element contamination to samples by glass materials, we used acid-washed erlens and bottles and disposable polypropylene tubes and pipets (Tubes 14 ml PP Falcon 352059 and pipets 25 ml Falcon 357535). Wild type and Δ*rpoS Salmonella* strains (VF6910 and VF8158 respectively, S1 Table) were grown in LB at 37°C for 18h. For complementation experiments, both strains harboring the vector pACYC184 and the cloned *rpoS* gene on pSTK4 (S1 Table) were grown in LB supplemented with chloramphenicol at 37°C for 18h. Three biological replicates of each strain were used. Twenty-eight ml of each culture were centrifuged in 50-ml polypropylene tubes (Sarstedt 62-547-254) for 10 min at 4°C and 6,300xg. Cell pellets were washed twice with distilled water containing 1 mM EDTA (pH 8) to chelate extracellular traces of metals, and centrifuged again. Cells were resuspended in 2.8 ml distilled water with EDTA (pH 8) 1 mM. The OD_600_ was measured and the number of viable cells was estimated by plating serial dilutions on LB. Each of the three independent biological replicates was subsequently treated in duplicates. Cell suspensions were each transferred in two pre-weighed microtubes (1.3 ml per tube, eppendorf 033297), centrifuged 10 min at 4°C and pellets were dried in a heat block overnight at 65°C. Dried cell pellets were digested in 2 ml of nitric acid (67%) and 4 ml H_2_O_2_ (30%) in pre-weighed 50-ml polypropylene tubes (DigiTube®, SCP Sciences, France) previously checked for element contamination. The tubes were left at room temperature for 24 hours and then evaporated, at 60°C for ~2 h and at 95°C for ~2 h, to near dryness to a final volume of 100 *μ*l, and then diluted to 7.5 ml in 0.5 N HNO_3_. Ultrapure reagents were used (Normatom grade, VWR, France for HNO_3_, and ANALAR Normapur grade, VWR, France for H_2_O_2_). Digestion blanks were run to check for contamination. Major (Na, Mg, Al, K) and trace element concentrations (Ti, V, Cr, Mn, Fe, Co, Ni, Cu, Zn, As, Se, Sr, Mo, Ag, Cd, Sb, Ba, Tl, Pb) were determined in mineralized solutions and in the growth medium using an inductively coupled plasma quadrupolar mass spectrometer (ICP-QMS) (X-Series, CCT II+ Thermo-electron, France). Internal standards (Re, Rh and In; SPEX, SCP Science, France) were used to correct for instrumental drift and plasma fluctuation. To limit interferences, analysis was performed using a collision cell technology (CCT), which introduces a supplementary gas mixture of H_2_ (7%) and He (93%) for the determination of V, Cr, Mn, Fe, As, Se, Ag, and Sb concentrations. A certified river water sample (SRM 1640a, NIST, Gaithersburg, USA) was repeatedly analyzed to check for data quality for all elements, except for Ti for which no certified values are provided. The SRM 1640a was 10-fold and 100-fold diluted to fit sample concentration range. The measured concentrations fall with 5% of the certified values for all elements, except Al (6.5%), As (7%) and Sb (8%).

### Statistical analysis

Student’s t-test was performed for pairwise comparisons. Values were presented as means ± standard error of the mean (SEM). Differences were considered significant when p ≤ 0.05.

## Results and discussion

### σ^S^ activates transcription of the *ryhB1* and *ryhB2* sRNA genes

**Fig 1:**
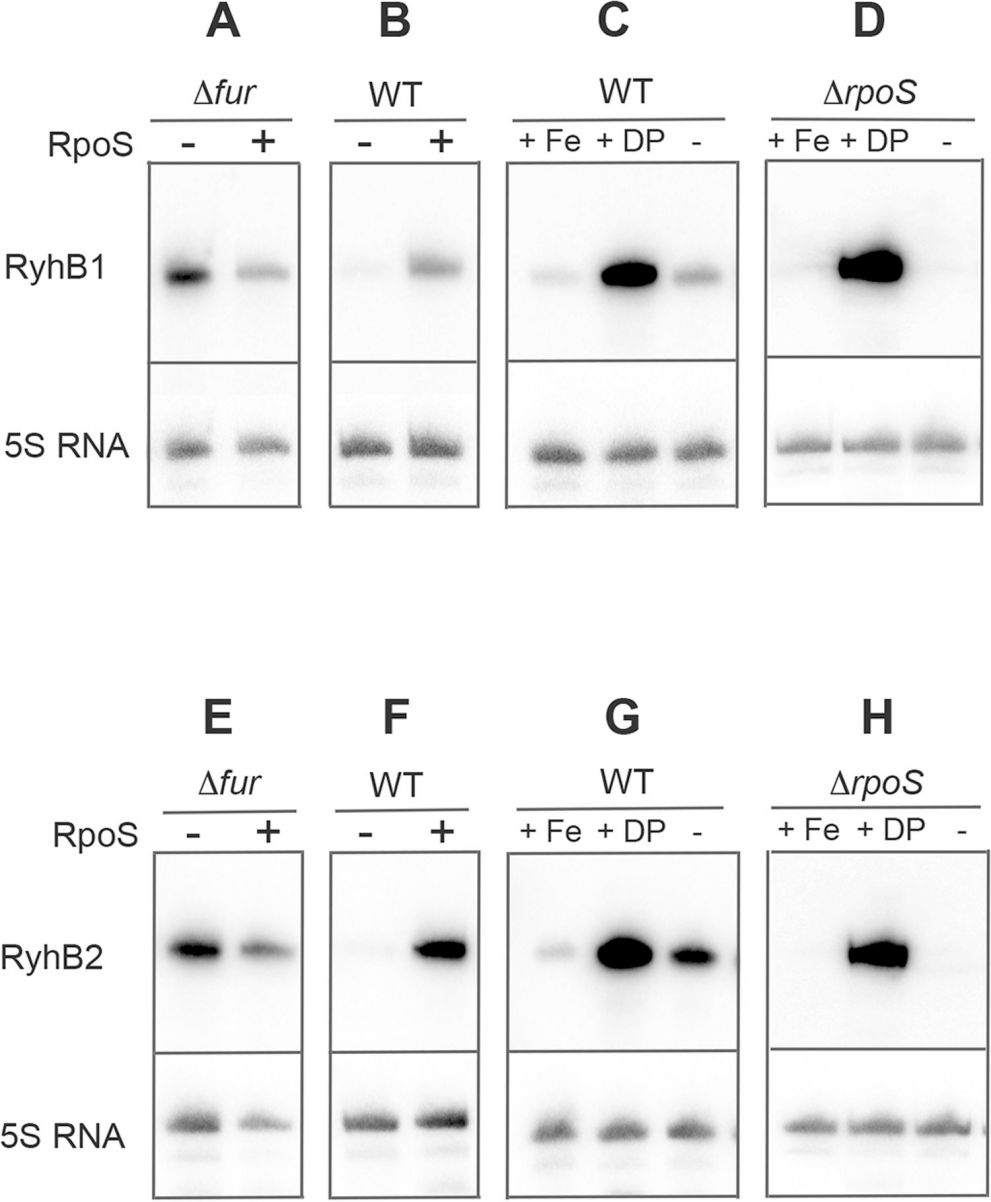
Regulation of *ryhB1* and *ryhB2* by σ^S^ in *Salmonella*. The RyhB1 and RyhB2 sRNAs (of 96 and 98 nucleotides, respectively) were detected in Northern experiments as previously reported [5]. Blots were stripped and re-probed with 5S RNA probe to confirm loading of equal quantities of total RNA of the strains grown to stationary phase for 18 h at 37°C. (A, E) Effect of the Δ*rpoS* mutation on *ryhB1* and *ryhB2* expression was assessed in a Δ*fur* genetic background. (B, F) Control experiments showing the negative effect of the Δ*rpoS* mutation on *ryhB1* and *ryhB2* expression [5]. (C, D, G, H) Detection of the sRNAs in the wild-type and Δ*rpoS* strains grown in LB supplemented or not with FeCl_2_ 100 *μ*M and 2,2’-dipyridyl (DP) 100 *μ*M. Relative quantification of bands intensity (normalized to the 5S RNA) indicated that the Δ*rpoS* mutation decreased by about ten-fold the amounts of RyhB1 and RyhB2 detected in the wild-type strain, as previously reported [5]. In contrast, in the Δ*fur* background, the Δ*rpoS* mutation had no major impact on the amount of RyhB2 and increased the amount of RyhB1 by about three-fold. Blocking Fur-mediated repression by DP increased the expression levels of both sRNAs by more than 10-fold in the Δ*rpoS* mutant. In the wild-type strain, the impact of DP was stronger on RyhB1 than RyhB2 production (10 and 3.5 -fold, respectively).

**Fig 2:**
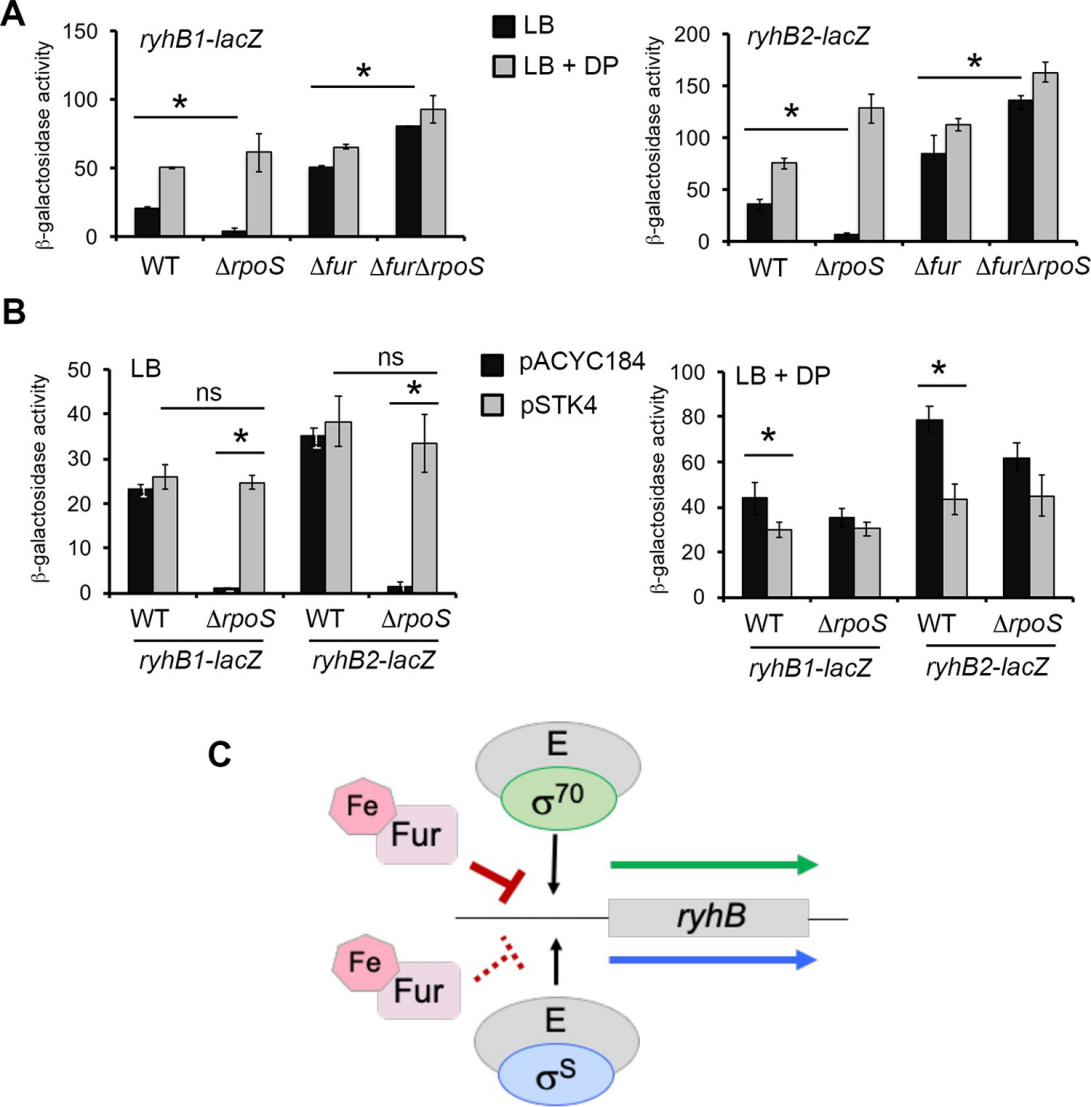
Expression of *ryhB1-lacZ* and *ryhB2-lacZ* transcriptional fusions in *Salmonella*. (A) Expression of the *ryhB1-lacZ* and *ryhB2-lacZ* transcriptional fusions was followed in *Salmonella* wild type, Δ*rpoS*, Δ*fur and* Δ*rpoS*Δ*fur* strains (S1 Table) grown 18 h in LB supplemented or not with 2,2’-dipyridyl (DP) 100 *μ*M. (B) Empty vector pACYC184 and plasmid pSTK4 carrying the *rpoS* gene (S1 Table) were used in complementation experiments. (A, B) Bar graphs represent the mean β-galactosidase activity, and error bars represent standard error of the mean of at least three independent experiments (* p<0.05, ns not significant). (C) Schematic illustration of regulation of *ryhB* genes by σ^S^ and Fur. In stationary phase of growth, Eσ^S^ and Eσ^70^ RNAP compete for binding to the *ryhB1* and *ryhB2* promoters. When iron is available, Fur-Fe^2+^ dimers bind to the *ryhB* promoter regions and repress σ^70^-dependent transcription. Eσ^S^ is less sensitive to Fur-mediated repression than Eσ^70^ and allows transcription of *ryhB1* and *ryhB2*. The exact mechanisms by which Eσ^S^ escapes Fur-mediated repression is unknown. Since the Fur binding sites overlap the promoter regions (Fig 3A), it is likely that Fur inhibits *ryhB1* and *ryhB2* transcription by occluding the promoters to prevent Eσ^70^ binding. Eσ^S^ might be more efficient than Eσ^70^ to compete with Fur for binding to the Fur box/ -10 region. However, the possibility that counter-silencing involves a structural change at the DNA level that allows Eσ^S^ binding despite the presence of Fur cannot be excluded.

In previous RNA sequencing experiments using wild-type and Δ*rpoS* strains of *S*. Typhimurium ATCC14028 grown to stationary phase in LB rich medium, the RyhB1 and RyhB2 sRNAs were detected in lower amounts in the Δ*rpoS* mutant than in the wild-type strain [5]. The positive effect of σ^S^ on these sRNAs levels was also observed in northern experiments [5,14] (Fig 1B and 1F). We used transcriptional *lacZ* fusions located downstream of the *ryhB1* and *ryhB2* promoters [14] to demonstrate that this σ^S^ control operates at the transcriptional level (Fig 2A). Introduction in the Δ*rpoS* mutant of plasmid pSTK4, carrying the *Salmonella rpoS* gene, restored wild-type levels of expression of *ryhB1-lacZ* and *ryhB2-lacZ* (Fig 2B), thus confirming that σ^S^ activates transcription of both sRNAs. Unexpectedly however, the Δ*rpoS* mutation did not abolish, and even slightly increased the amounts of RyhB1 and RyhB2 and the expression levels of the *ryhB1-lacZ* and *ryhB2-lacZ* fusions in the Δ*fur* mutant (Figs 1A, 1E and 2A). In addition, expression levels of the RyhB1 and RyhB2 sRNAs (Fig 1C, 1D, 1G and 1H) and the *ryhB1-lacZ* and *ryhB2-lacZ* fusions (Fig 2A) were increased, in both the wild-type strain and Δ*rpoS* mutant, by 2,2-dipyridyl (DP) that sequesters free iron and consequently likely impairs Fur activity. Altogether, these results were consistent with a model where σ^S^ is required for *ryhB1* and *ryhB2* transcription under Fur repressing conditions only (Fig 2C). In our global analyses, the Δ*rpoS* mutation had no effect on the abundance of the *fur* transcripts [5] and Fur protein [7], indicating that σ^S^ does not alleviate *ryhB1* and *ryhB2* repression by decreasing Fur expression.

### σ^S^ counteracts Fur mediated repression of *ryhB1* and *ryhB2* transcription

**Fig 3:**
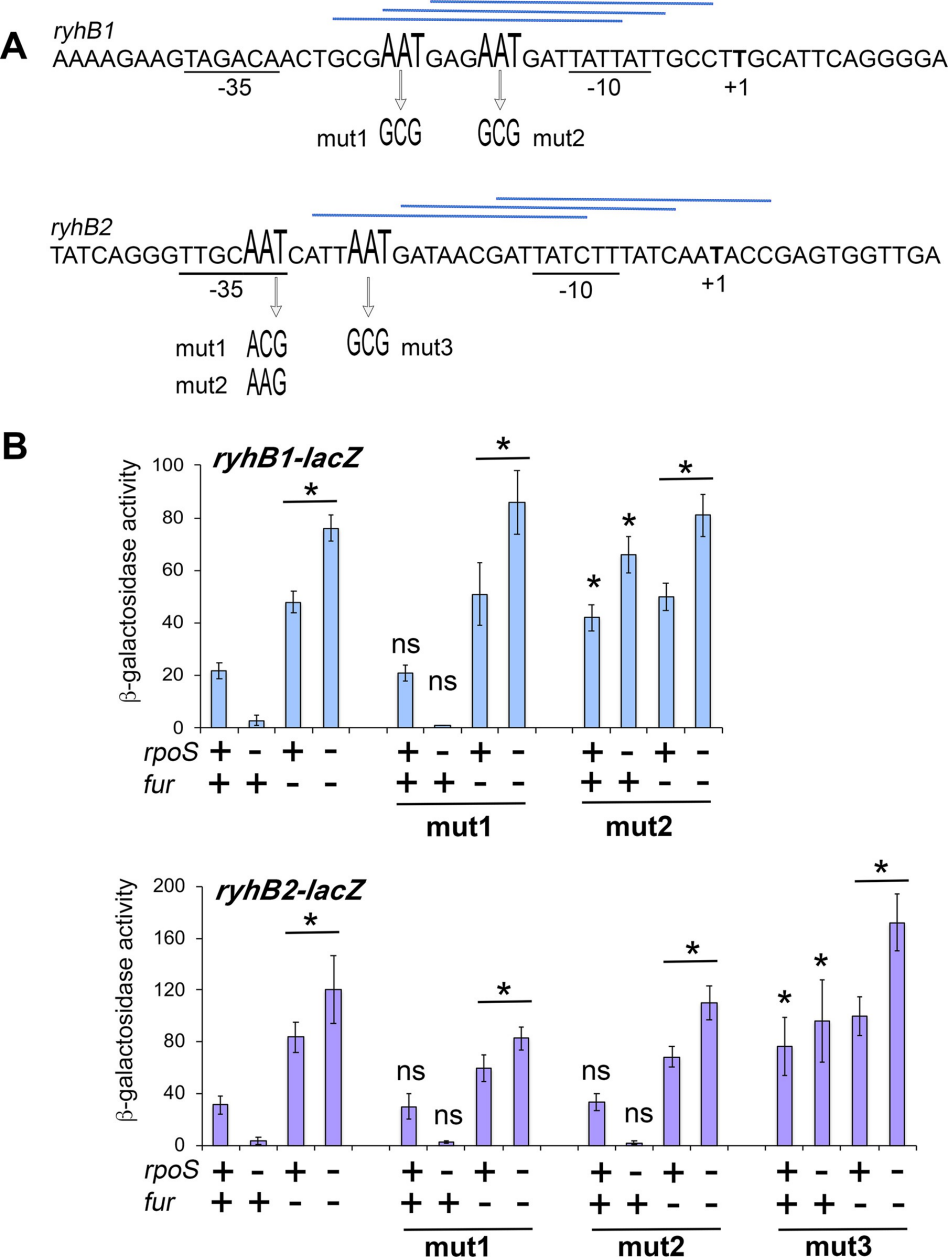
Effect of mutations in Fur binding motifs on regulation of the *ryhB1-lacZ* and *ryhB2-lacZ* fusions. (A) DNA sequences corresponding to the 5’ end and upstream regions of the *ryhB1* and *ryhB2* sRNAs genes are shown. The -10 and -35 promoter regions are underlined. The possible Fur binding sites that match the Fur consensus sequence (gataatgataatcattatc) are indicated by blue lines above the sequences of *ryhB1* and *ryhB2* [14]. Mutations constructed in AAT motifs are shown (see text for details). (B) Expression of the chromosomal *ryhB1-lacZ* and *ryhB2-lacZ* fusions carrying or not the indicated mutations was assessed in *Salmonella* wild type, Δ*rpoS*, Δ*fur and* Δ*rpoS*Δ*fur* strains (S1 Table) grown 18 h in LB. Bar graphs represent the mean β-galactosidase activity, and error bars represent standard error of the mean of at least three independent experiments (* p<0.05, ns not significant). The finding that mut3 relieves Fur repression of *ryhB2* suggests that Fur binds to the more distal predicted binding site.

The *ryhB1* and *ryhB2* promoters show typical features of σ^70^ dependent promoters [14] which can also be recognized by σ^S^ [1,2] (Fig 3A). The Fur-binding site of regulated promoters contains a consensus of three imperfect adjacent hexamers 5’-GATAAT-3’ [28] and alignment of Fur-regulated genes in *S*. Typhimurium has underlined the importance of the AAT motifs in Fur binding [29]. Consensus binding sites for Fur overlap the *ryhB1* and *ryhB2* promoter regions [14] (Fig 3A). Mutations in AAT motifs located upstream of the -10 sequence in the predicted Fur binding sites were introduced in the promoter regions of the *ryhB1-lacZ* and *ryhB2-lacZ* chromosomal fusions to assess σ^S^ and Fur regulation. Mutations were also introduced in the AAT motif present in the -35 region of *ryhB2*.The mut1 mutation did not affect the expression level and regulation of the *ryhB1-lacZ* fusion (Fig 3B). In contrast, the mut2 mutation abolished Fur repression of *ryhB1-lacZ* and the expression level of *ryhB1*_*mut2*_*-lacZ* was slightly improved in the absence of σ^S^ (Fig 3B). Mut3 was the only mutation preventing Fur repression of the *ryhB2-lacZ* fusion and expression of *ryhB2*_*mut3*_*-lacZ* did not require σ^S^ (Fig 3B). Altogether, these data suggest that σ^S^ favors *ryhB1* and *ryhB2* transcription by directly counteracting Fur-mediated repression. Since Eσ^S^ binds to promoters and is sensitive to repressors in a manner distinct from Eσ^70^ [1], it is possible that Eσ^S^, but not Eσ^70^, competes efficiently with Fur for promoter binding or is productive to some extent in the presence of Fur, and allows transcriptional initiation from the *ryhB1* and *ryhB2* promoters (Fig 2C). Levels of *ryhB1* and *ryhB2* expression were slightly higher in the Δ*fur*Δ*rpoS* mutant than in the Δ*fur* strain (Figs 1A, 1E, 2A and 3B) and the *rpoS* gene on pSTK4 reduced the expression level of *ryhB2-lacZ* (and to a lesser extent *ryhB1-lacZ*) in the presence of DP (Fig 2B). These results suggest that σ^S^ is less efficient than σ^70^ for *ryhB* transcription when Fur repression is eliminated. This hypothesis is consistent with previous findings that σ^S^ can directly repress gene expression by competing with σ^70^ binding at some promoters [6,30,31].

### σ^S^ alleviates repression of genes involved in iron and manganese metabolism

**Fig 4:**
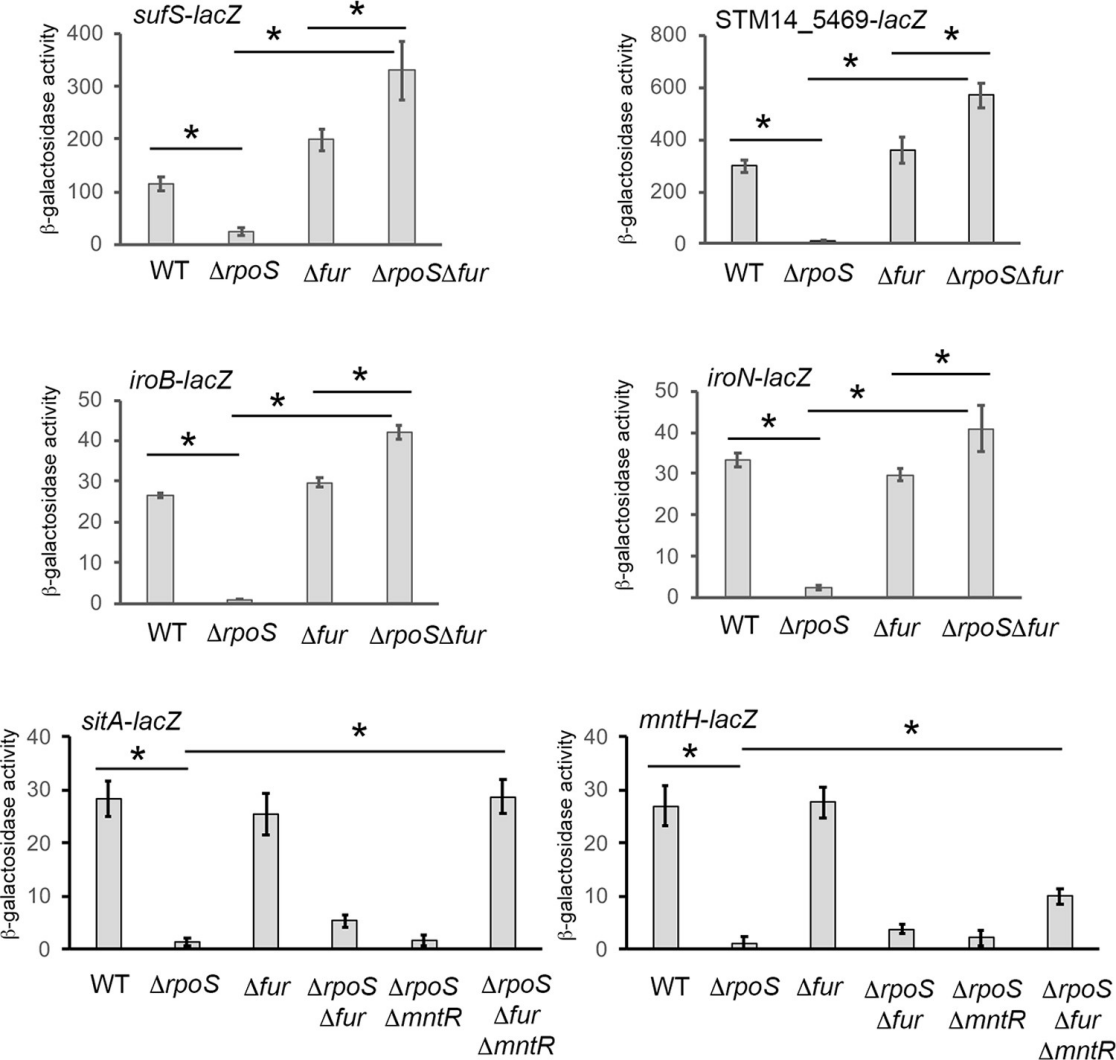
σ^S^ alleviates repression of genes involved in iron and manganese metabolism. Expression of the indicated *lacZ* fusions was assessed in the wild type Δ*rpoS*, Δ*fur* and Δr*poS*Δ*fur* strains grown for 18 h in LB at 37°C. For the *sitA-lacZ* and *mntH-lacZ* fusions, effect of the Δ*mntR* mutation was also determined. Bar graphs represent the mean β-galactosidase activity, and error bars represent standard error of the mean of at least three independent experiments (* p<0.05).

Among genes that are strongly activated by σ^S^ in ATCC14028 [5], some are repressed by Fur [8,32]. We thus addressed the possibility that σ^S^ counteracts Fur repression of additional genes, besides *ryhB1* and *ryhB2*. The *suf* genes encode an alternative system for iron-sulfur clusters assembly repressed by Fur [33,34] and were strongly activated by σ^S^ in our transcriptomic and proteomic analyses [5,7]. The *sufS-lacZ* fusion was up- and down- regulated in the Δ*fur* and Δ*rpoS* strains, respectively, but its expression in the Δ*fur* mutant did not require σ^S^ (Fig 4). Similarly, expression of the *lacZ* fusions in the *iroBCDE* operon and *iroN* gene, involved in biosynthesis and utilization of the salmochelin siderophore [35], and the STM14_5469/*yjjZ* gene, involved in tolerance to biofuels and antibiotics [36–38] was dependent on σ^S^ in the presence of Fur only (Fig 4). These data suggest that σ^S^ counteracts Fur repression of these genes, as it was observed for *ryhB1* and *ryhB2*.

The *sitABCD* and *mntH* genes, encoding manganese transporters, are also repressed by Fur [39,40] and activated by σ^S^ [5], but their expression was still dependent on σ^S^ in the absence of Fur (Fig 4). Since these genes are also repressed by the manganese-responsive repressor MntR [39,40], their expression was also assessed in a Δ*mntR* background. The *sitA-lacZ* fusion was expressed to similar levels in the wild-type strain and the Δ*rpoS*Δ*fur*Δ*mntR* mutant (Fig 4), indicating that σ^S^ was dispensable only when both repressors were absent. The expression level of the *mntH-lacZ* fusion increased in the Δ*rpoS*Δ*fur*Δ*mntR* mutant compared to that in the Δ*rpoS* strain, but was lower than that in the wild-type strain (Fig 4). This result suggests that, even in the absence of Fur and MntR, σ^S^ is necessary, directly or indirectly, for *mntH* expression. σ^S^ might alleviate repression of *mntH* by a third unknown repressor molecule or favor the production of an activator. Altogether, these data demonstrated that σ^S^ allows expression of genes involved in iron and manganese metabolism under environmental conditions where their transcription by σ^70^ is repressed. Of note, the regulatory interplay between Fur and σ^S^, highlighted here, is not a general phenomenon since several genes sensitive to Fur repression are not activated by σ^S^ in the SalCom database [8] (S2 Fig).

### Effects of σ^S^ on the *Salmonella* ionome in stationary phase

**Fig 5:**
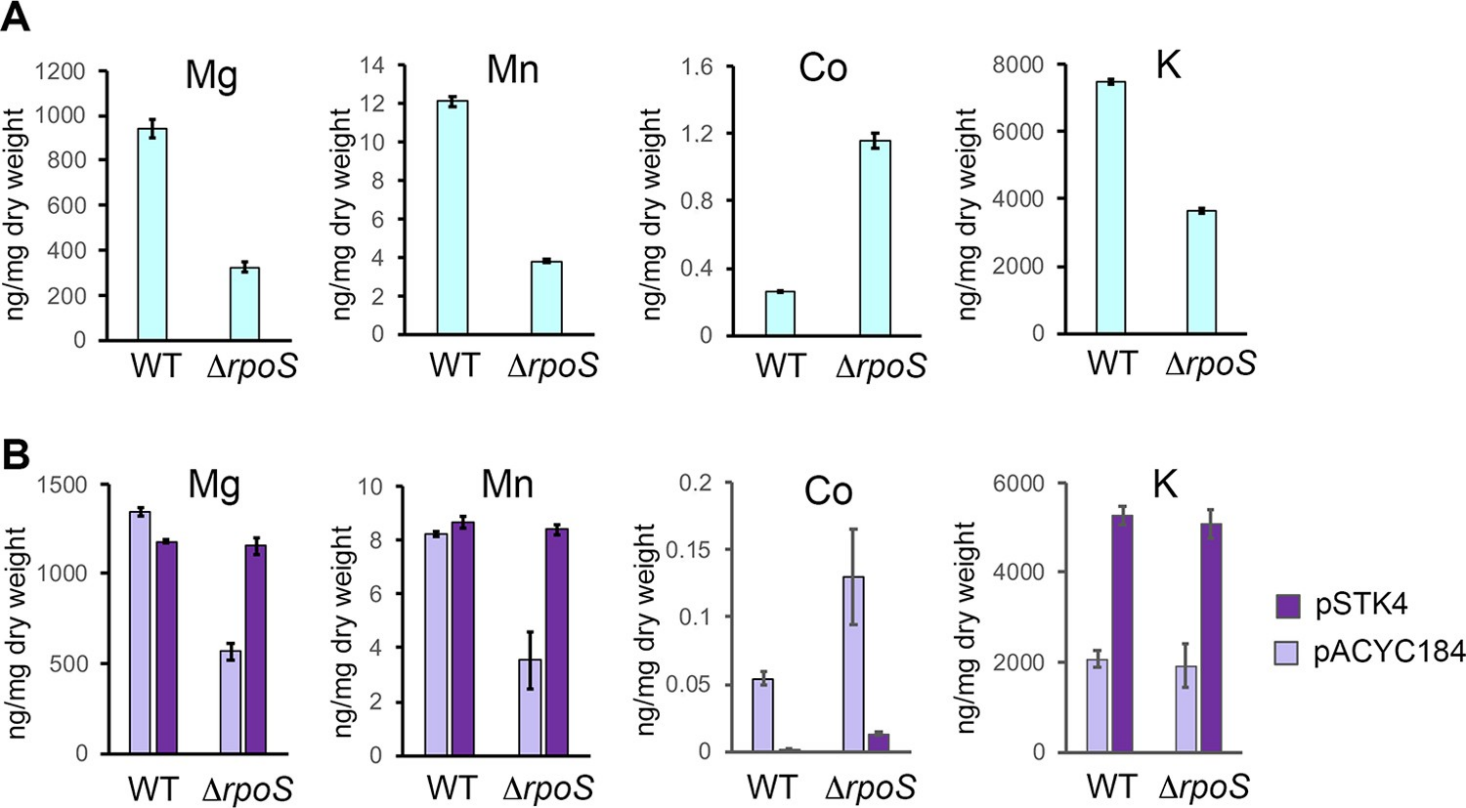
Effect of the Δ*rpoS* mutation on the *Salmonella* ionome in stationary phase. Quantification of cell-associated concentration of elements in *Salmonella*, grown to stationary phase in LB rich medium, was performed by inductively coupled plasma mass spectrometry (see Methods). Three biological replicates were analyzed in duplicate each. A complete set of data is provided in S1 Dataset. (A) Data are shown for element contents showing significant differences (p<0.05, fold-change > 2) between the wild type strain (WT, VF6910) and the Δ*rpoS* mutant (VF8158): Magnesium (Mg), manganese (Mn), cobalt (Co) and potassium (K). (B) Complementation experiments with the cloned *rpoS* gene on pSTK4. The empty vector pACYC184 was used as a control. Error bars display the standard error of the mean for the three independent replicates (A, B).

The observed control by σ^S^ of genes involved in iron and manganese metabolism prompted us to assess the impact of σ^S^ on the *Salmonella* ionome. Inductively coupled plasma mass spectrometry (ICP-MS) was used to compare levels of cell-associated metals in the wild-type and Δ*rpoS* strains grown to stationary phase in LB (Fig 5A and S1 Dataset experiment 1). The Δ*rpoS* mutation had no significant effect (p>0.05) on the cellular concentration of Se, Ni, Cu, Sr, Ba and Ti and modest effects on the cellular concentration of Na, V, Zn, Fe, As, Mo, Cd and Tl (fold change < 2, p<0.05, S1 Dataset experiment 1). In contrast, a marked effect of the Δ*rpoS* mutation (fold change > 2, p<0.001) was observed on the cell-associated concentration of cobalt (Co), manganese (Mn), magnesium (Mg) and potassium (K) (Fig 5A, S1 Dataset experiment 1).

To validate these effects of σ^S^ on the ionome, a complementation experiment was conducted (Fig 5B and S1 Dataset experiment 2). The cloned *rpoS* gene on pSTK4 was able to restore wild-type levels of Mn, Mg and Co in the Δ*rpoS* strain, thus confirming the role of σ^S^ in the control of the cell-associated amount of these cations. The potassium contents of the wild-type strain and Δ*rpoS* mutant harboring pSTK4 were similar and significantly higher (fold change >2, p<0.001) than that of wild-type and Δ*rpoS* strains harboring the empty vector (Fig 5B and S1 Dataset experiment 2). These data were consistent with a positive effect of σ^S^ on the cell-associated concentration of potassium. However, the potassium amount was not significantly different (fold change 0.96, p> 0.1) between the Δ*rpoS* mutant and the wild-type strain harboring pACYC184, and was even lower than that in the absence of plasmid (Fig 5A and 5B). The potassium concentration was similar in the batches of LB used in experiments 1 and 2 (S1 Dataset). One possibility is that the presence of pACYC184 (or the *tetA* gene which is inactivated in pSTK4 by insertion of *rpoS*) impairs K^+^ fluxes thereby masking the effect of σ^S^. In conclusion, σ^S^ modulates the amount of manganese, magnesium, cobalt and likely potassium associated with quiescent cells.

### Regulation by σ^S^ of genes involved in ions trafficking

A positive effect of σ^S^ on the total cell concentration of manganese was consistent with our finding that σ^S^ activates transcription of the *mntH* and *sitABCD* genes [5] (Fig 4), even though we cannot exclude the contribution of transport systems for other metals able to accommodate Mn. Mn is a cofactor for several enzymes in bacteria and can contribute to the catalytic detoxification of reactive oxygen species (ROS) [39,40]. Increased cell-associated Mn concentrations could favor the activity of enzymes requiring Mn^2+^ as a cofactor and involved in metabolism and protection against oxidative stress. In addition, as previously suggested [5], in stationary phase of growth, Mn^2+^ might replace the more reactive Fe^2+^ ion in iron-containing enzymes to reduce oxidative damage to these proteins [40].

**Fig 6:**
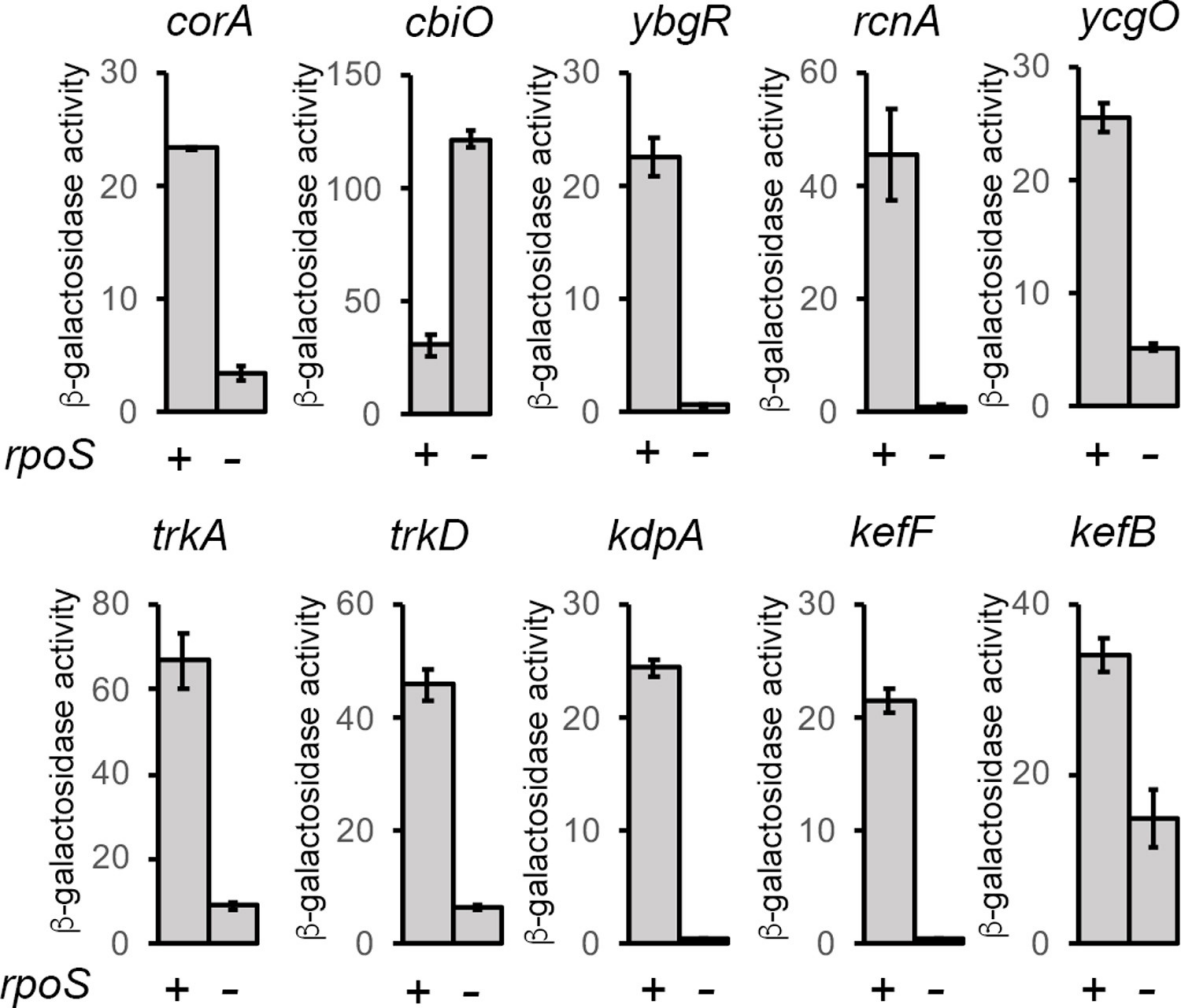
Expression of *lacZ* fusions in genes involved in magnesium, cobalt and potassium trafficking. Expression of *lacZ* fusions in genes encoding potassium transport (TrkA, TrkD, KdpA) and efflux (KefCF, KefB) systems, the potassium/proton antiporter YcgO, the magnesium transporter CorA and proteins involved in cobalt import (CbiO) and efflux (RcnA, YbgR) was assessed in the wild type and Δ*rpoS* strains grown for 18 h in LB at 37°C. Bar graphs represent the mean β-galactosidase activity, and error bars represent standard error of the mean of at least three independent experiments. The effect of the Δ*rpoS* mutation was significant in all cases (p<0.001).

The ICP-MS analysis also revealed a positive effect of σ^S^ on the cell-associated concentration of magnesium. In contrast to Mn^2+^ that can be transported by transport systems for other cations [41], the chemical properties of Mg^2+^ suggest that proteins mediating Mg^2+^ transport have unusual properties [42,43]. *Salmonella* imports magnesium *via* three known transporters, MgtA and MgtB produced under conditions of magnesium starvation and CorA, expressed under various growth conditions and able to perform Mg^2+^ import and efflux [43–46]. In our global transcriptomic analysis in LB, the *mgtA* and *mgtB* genes were expressed to similar and very low levels in the wild-type and Δ*rpoS* strains [5], likely because the magnesium concentration in LB is high enough (S1 Dataset) to prevent activation of these genes by the PhoP-PhoQ system [43,44]. In contrast, the *corA* gene was downregulated in the Δ*rpoS* mutant [5], a result that was confirmed by using a transcriptional *corA-lacZ* fusion (Fig 6). A reduced expression level of *corA* in the Δ*rpoS* strain may thus contribute to lower the magnesium content of the mutant, compared to the wild-type strain (Fig 5). Nevertheless, potential differences between the two strains in their membrane composition and/or ribosomes and ATP contents, which represent important reservoirs of magnesium [43,44,47,48], have also to be considered.

The potassium level of quiescent cells is also likely positively controlled by σ^S^. Potassium is the major monovalent cation in the bacterial cytoplasm and its concentration is regulated through the activity of a number of different transport and efflux systems [49–51]. Some of the corresponding genes were down regulated in the Δ*rpoS* mutant in our global analyses [5,7] and this σ^S^ control was validated by using transcriptional *lacZ* fusions in these genes (*ycgO*, *trkA*, *trkD*, *KdpA*, *kefF*, *kefB*, Fig 6). Consistent with a role of σ^S^ in potassium homeostasis, it has been suggested that the small σ^S^ -dependent protein YgaU/Kbp [5,7] is a cytoplasmic K^+^ sensor regulating potassium homeostasis in *E*. *coli* [52]. In addition, K^+^ stimulates σ^S^ activity [1,53].

In contrast to the other cations, cobalt was accumulated in the Δ*rpoS* mutant compared to the wild-type strain (Fig 5). Cobalt is a trace metal in extracellular media [54–56] including LB (S1 Dataset). In *Salmonella*, the *cbiMNQO* operon, located amongst the vitamin B12 biosynthesis genes, encodes a high affinity cobalt uptake system [54,55,57]. Transport systems for other metals, such as CorA, can also import cobalt [54,55,57] but are likely inefficient in LB where cobalt concentration is low (S1 Dataset). In our global proteomics profiling experiments [7], we noticed that the ATP-binding protein CbiO showed increased abundance in the Δ*rpoS* mutant of *Salmonella*, compared to the wild-type strain. The negative effect of σ^S^ on the CbiO relative protein levels was confirmed by using a translational *cbiO-lacZ* fusion (Fig 6). Two genes are annotated as putative efflux systems for cobalt in ATCC14028, STM14_0882/*ybgR* that encodes a zinc exporter ZitB [58] and STM14_3652/*yohM*/*rcnA* encoding an efflux protein for cobalt and nickel in *E*. *coli* [59,60]. Transcriptional *lacZ* fusions in both genes were positively controlled by σ^S^ (Fig 6). These data suggest that σ^S^ limits cobalt accumulation in quiescent cells even when cobalt is present at very low extracellular concentrations. Co^2+^, either as a cofactor or associated with vitamin B12, is required for many biological functions but it can also be toxic due to non-specific interaction with proteins or DNA, the formation of reactive oxygen species and the competition with iron which affects the biogenesis of iron-sulfur clusters [54,56,61]. Quiescent cells may be very susceptible to oxidative damages and the Co effects, thereby requiring a tight control of cobalt accumulation by σ^S^.

### σ^S^ is required for optimal regrowth of quiescent cells in LB depleted for magnesium

Future experiments are required to determine whether the transcriptional effects of σ^S^ on genes involved in ion trafficking reported in this study are implicated in the observed modulation of the ionome by σ^S^ and whether additional effects of σ^S^ on unspecific transport, efflux and storage systems are involved. Nevertheless, the regulation by σ^S^ of the cell-associated levels of Co^2+^, Mn^2+^, Mg^2+^ and possibly K^+^ suggest that a tight control of uptake and availability of these cations might be critical for quiescent bacteria, as an imbalance in homeostasis of these cations may be deleterious for their survival or regrowth potential.

**Fig 7:**
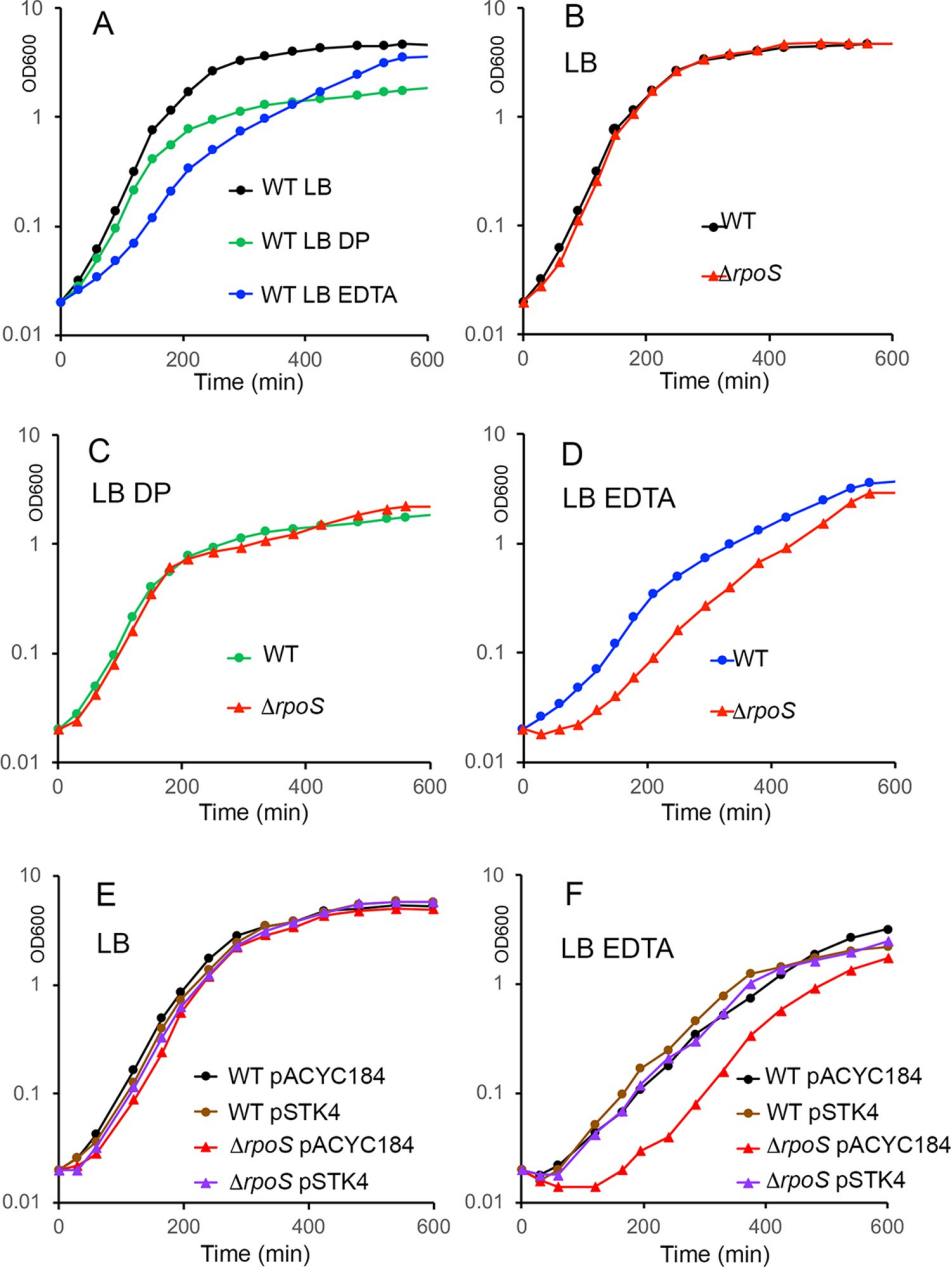
The metal ion chelator EDTA extends the lag phase of the Δ*rpoS* mutant. (A, B, C, D) Kinetics of growth of the wild-type strain (WT, VF6910) and the Δ*rpoS* mutant (VF8158) was followed in LB supplemented or not with EDTA 2 mM or DP 200 *μ*M. (E, F) The empty vector pACYC184 and plasmid pSTK4 carrying the *rpoS* gene were used in complementation experiments. The growth phase was determined by the measurement of culture turbidity at OD 600 nm. Similar results were obtained with the two biological replicates of each strain that were tested (see S3A and S3B Fig for the second series of biological replicates). Similar results were also observed using an independent Δ*rpoS* construct (VFC331, S4 Fig).

**Fig 8:**
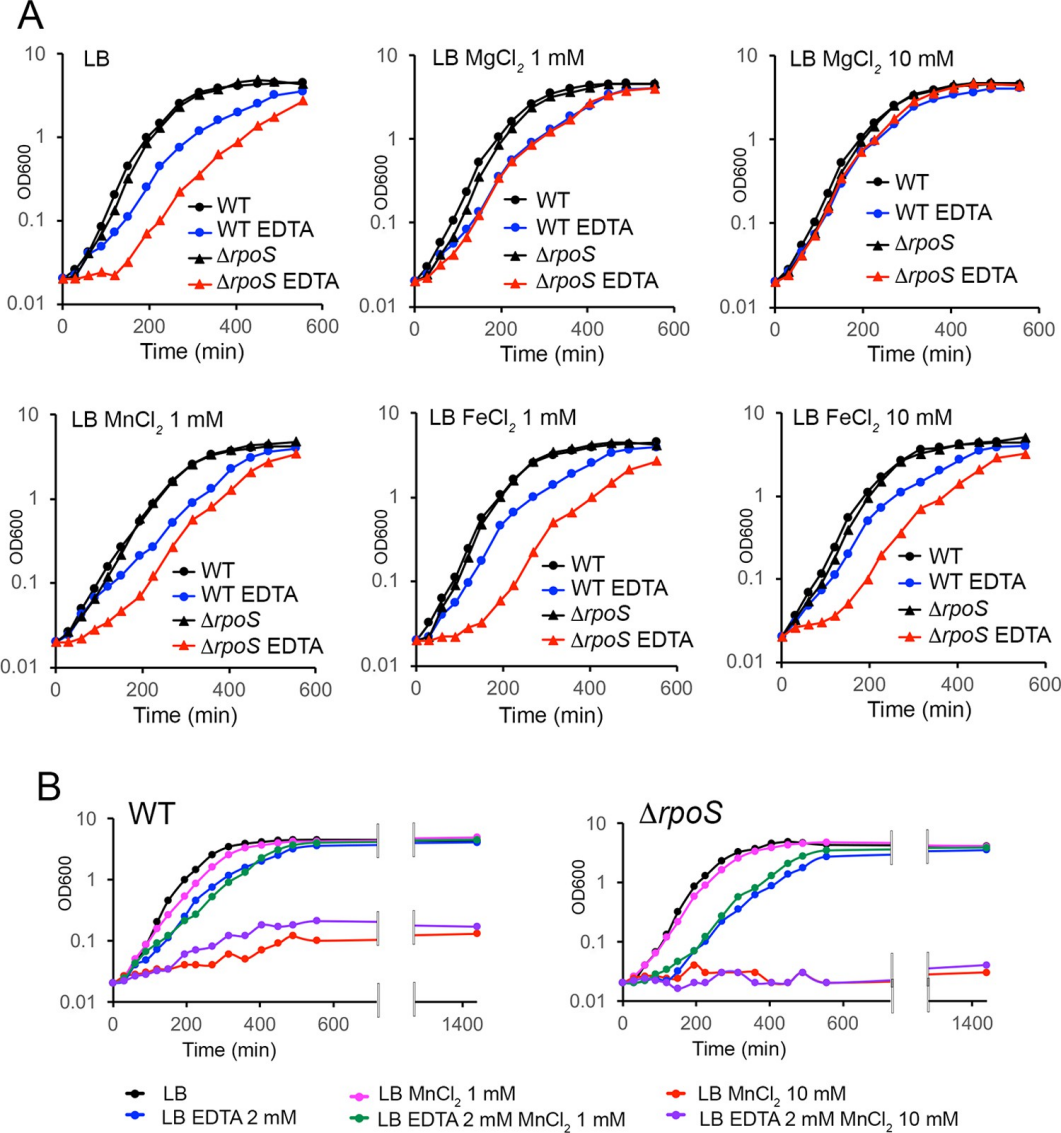
Magnesium abolishes the effects of EDTA in add-back experiments. Kinetics of growth of the wild-type strain (WT VF6910) and the Δ*rpoS* mutant (VF8158) was followed in LB supplemented or not with EDTA 2 mM and the indicated metal ions. The growth phase was determined by the measurement of culture turbidity at OD 600 nm. The experiments were repeated twice with similar results (see S5 Fig for a repeat experiment).

As a first step to address this issue, the regrowth of the wild-type and Δ*rpoS* strains was examined in LB supplemented either with EDTA, a metal ion chelating agent, or with the iron chelating agent DP. The growth curves of the wild-type and Δ*rpoS* strains were similar in LB and, for both strains, the entry into stationary phase occurred at lower optical density in the presence of DP (Fig 7A–7C). Interestingly, even though similar numbers of CFU of the wild-type and Δ*rpoS* strains (2.9 10^7^ and 2.6 10^7^ CFU/mL, respectively) were inoculated into fresh LB medium supplemented with EDTA, the lag phase of the Δ*rpoS* mutant was extended, compared to that of the wild-type strain (Figs 7D and S3A). This effect of the Δ*rpoS* mutation was complemented by the cloned *rpoS* gene on pSTK4 (Figs 7E, 7F and S3B) and was reproducibly observed using another Δ*rpoS* construct (S4 Fig). Add-back experiments showed that magnesium, but not manganese or iron, was able to abolish the effects of EDTA (Figs 8A and S5). Indeed, EDTA did not extend the lag phase of the Δ*rpoS* strain when MgCl_2,_ but not MnCl_2_ or FeCl_2_, was supplemented at 1 mM suggesting that σ^S^ was required for optimal regrowth in LB depleted for magnesium. In addition, when supplemented at 10 mM, MgCl_2_, but not FeCl_2_, alleviated the effect of EDTA on bacterial growth (Figs 8A and S5).

In contrast to magnesium, high concentration of manganese prevented bacterial growth (Fig 8B). It is likely that the toxic effects of manganese on growing cells result from unspecific interactions with transporters of other metal ions or with metallobiomolecules that are important for growth. Interestingly, it has been proposed that excess Mn impairs bacterial growth by competing for magnesium and/or iron [39,62–64].

## Conclusion

Even though more studies are required to dissect the molecular mechanisms underlying the σ^S^-effects on the *Salmonella* ionome and their impact at the physiological level, this study reveals that σ^S^ controls the homeostasis and/or usage of Mg, Mn, Co, K and Fe, thereby suggesting an impact of these cations on the fitness of quiescent cells. Our results pinpoint to the importance of magnesium for optimal regrowth of quiescent cells. The reduced magnesium content of the Δ*rpoS* mutant, compared to the wild type strain, correlates with a longer lag phase of the mutant when LB was depleted for magnesium. Magnesium concentration may be adjusted by σ^S^ in quiescent cells to offer optimal regrowth of cells under conditions of magnesium limitation.

Lag phase is a poorly understood stage of the bacterial growth cycle [65–67]. This period prepares bacteria for the replicative phase and is thus critical for competitive growth of bacteria and possibly antibiotic tolerance. The *Salmonella* lag phase in LB rich medium was shown to involve transient metal accumulation [65]. The cell-associated concentration of magnesium was maximal in mid-exponential phase of growth but did not change significantly during the lag phase [65]. In contrast, it was shown that iron, calcium, and manganese are accumulated during the lag phase whereas cobalt concentration is reduced [65]. It is tempting to speculate that the effects of σ^S^ on the *Salmonella* ionome and Fur-dependent regulation reported in the present study contribute to this phenomenon. Since σ^S^ counteracts Fur repression of several genes, it is likely that expression of these genes provides an advantage to non-actively growing *Salmonella* cells, either for long-term survival or for exit from dormancy under specific environmental conditions. Interestingly, the *sitABCD*, *mntH*, *iro* and *suf* genes are induced at the onset of the lag phase in LB [65]. It will be interesting to determine whether σ^S^ is involved in this regulation at the early lag phase and contributes to the accumulation of manganese and iron.

No effect of the Δ*rpoS* mutation was revealed on the cell-associated iron concentration in stationary phase, suggesting that the σ^S^/Fur interplay unraveled in this study does not affect the content, but rather modulates the usage, of iron in quiescent cells. In particular, stationary phase cells likely relay upon the alternative Suf machinery, rather than the housekeeping Isc system, for Fe-S cluster assembly, an hypothesis consistent with the findings that Suf is more resistant to oxidation than Isc and is functional under iron-limiting conditions [34]. Also, σ^S^ limits production of non-essential iron containing enzymes, such as the succinate dehydrogenase complex Sdh (directly at the promoter level and post-transcriptionally *via ryhB1* expression) and increases the production of iron storage proteins like Dps [5–7]. σ^S^ may also relieve repression of genes important for *Salmonella* regrowth in host and non-host iron limiting environments, such as the *iro* genes involved in production and utilization of siderophores [35]. Interestingly, siderophore production is associated with oxidative stress protection through iron sequestration or other mechanisms [68–71]. Accumulation of iron can promote the formation of reactive oxygen species through the Fenton reaction [72]. σ^S^ may affect iron usage to simultaneously control the intracellular level of free iron during the stationary phase and prepare the cell to a rapid accumulation of iron during the lag phase to support the replicative period and iron sequestration by the host during infection. This strategy would prevent oxidative damage while maintaining the regrowth potential and virulence of quiescent cells.

## Supporting information

S1 Fig Relative expression levels of sRNA genes in *Salmonella* wild-type and mutants. Heatmaps were recovered from the *Salmonella* SalCom database **(**http://bioinf.gen.tcd.ie/cgi-bin/salcom.pl?header_rotation=45;query=prpB;db=SalComRegulon_HL). As mentioned in Colgan *et al*. [8], “strains were grown either in Lennox broth to OD600 0.1 (EEP), 0.3 (MEP), 1.0 (LEP) 2.0 (ESP) and 2.0 + 6 h (LSP) or in the InSPI2 condition (slightly acidic pH and limitation of inorganic phosphate) which mimics aspects of the intra-macrophage conditions and induces expression of SPI2 Type 3 secretion system”.
(TIF)

S2 Fig Relative expression levels of Top 50 up-regulated genes in a Δ*fur* mutant. Heatmaps were recovered from the *Salmonella* SalCom database **(**http://bioinf.gen.tcd.ie/cgi-bin/salcom.pl?header_rotation=45;query=prpB;db=SalComRegulon_HL). As mentioned in Colgan *et al*. [8], “strains were grown either in Lennox broth to OD600 0.1 (EEP), 0.3 (MEP), 1.0 (LEP) 2.0 (ESP) and 2.0 + 6 h (LSP) or in the InSPI2 condition (slightly acidic pH and limitation of inorganic phosphate) which mimics aspects of the intra-macrophage conditions and induces expression of SPI2 Type 3 secretion system”.
(TIF)

S3 Fig The metal ion chelator EDTA extends the lag phase of the Δ*rpoS* mutant. Biological replicates of that in Fig 7.
(TIF)

S4 Fig The metal ion chelator EDTA extends the lag phase of the Δ*rpoS* mutant VFC331. Same experiment as in Fig 7A–7D, but using the Δ*rpoS* mutant VFC331.
(TIF)

S5 Fig Magnesium abolishes the effect of EDTA in add-back experiments. Independent repeat experiment of Fig 8.
(TIF)

S1 Table Strains used in this study. (DOC)

S2 Table Primers used in this study. (DOC)

S1 Dataset ICP-MS data. (XLSX)

S1 Raw images (PDF)
