# Supplementary figures and images for: The stress sigma factor σS/RpoS counteracts Fur repression of genes involved in iron and manganese metabolism and modulates the ionome of Salmonella enterica serovar Typhimurium
Source: PLoS One. 2022 Mar 31;17(3):e0265511. doi: 10.1371/journal.pone.0265511 (PMC8970401; doi:10.1371/journal.pone.0265511)

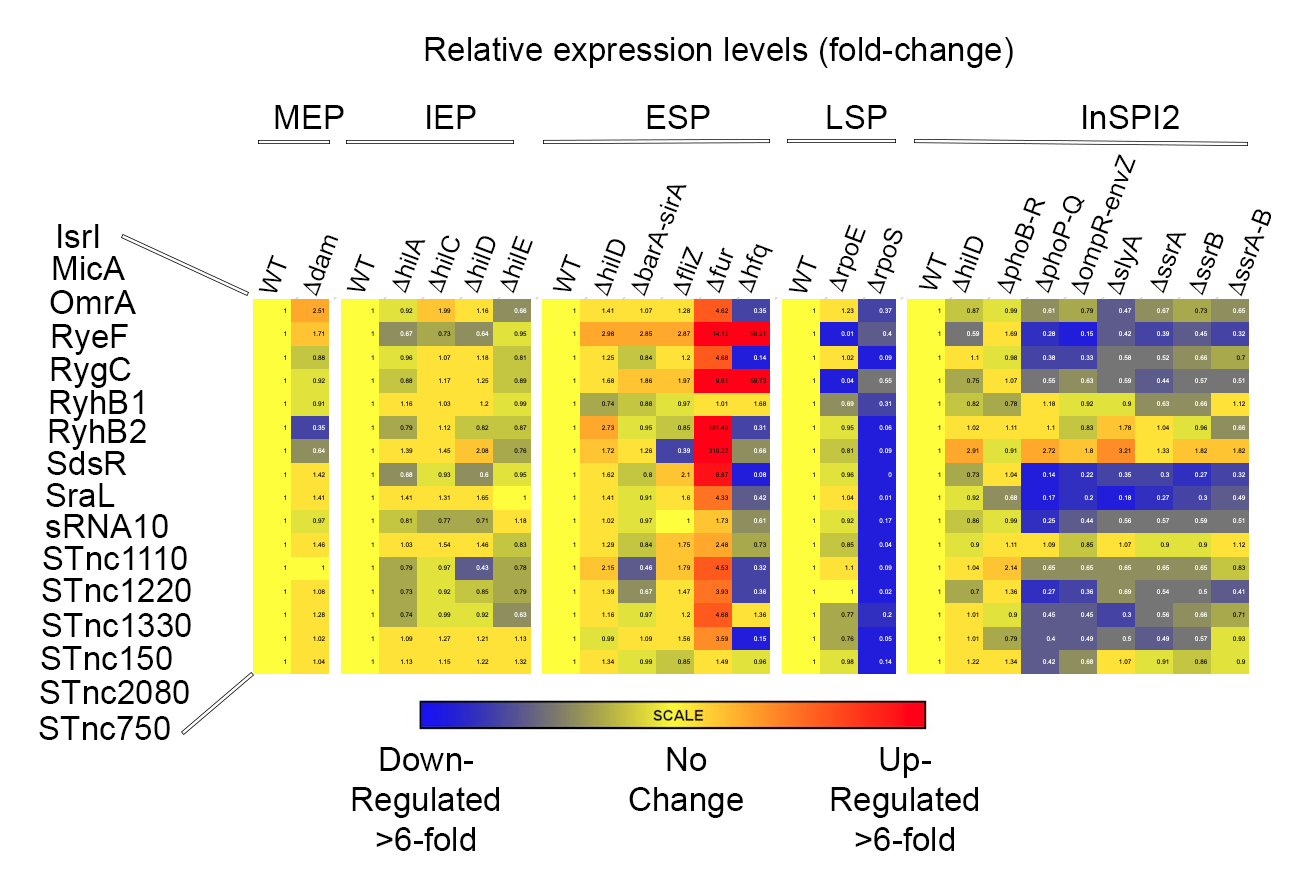

Supplement: S1 Fig — Heatmaps were recovered from the Salmonella SalCom database (http://bioinf.gen.tcd.ie/cgi-bin/salcom.pl?header_rotation=45;query=prpB;db=SalComRegulon_HL). As mentioned in Colgan et al. [8], “strains were grown either in Lennox broth to OD600 0.1 (EEP), 0.3 (MEP), 1.0 (LEP) 2.0 (ESP) and 2.0 + 6 h (LSP) or in the InSPI2 condition (slightly acidic pH and limitation of inorganic phosphate) which mimics aspects of the intra-macrophage conditions and induces expression of SPI2 Type 3 secretion system”. (TIF) [file pone.0265511.s001.tif]

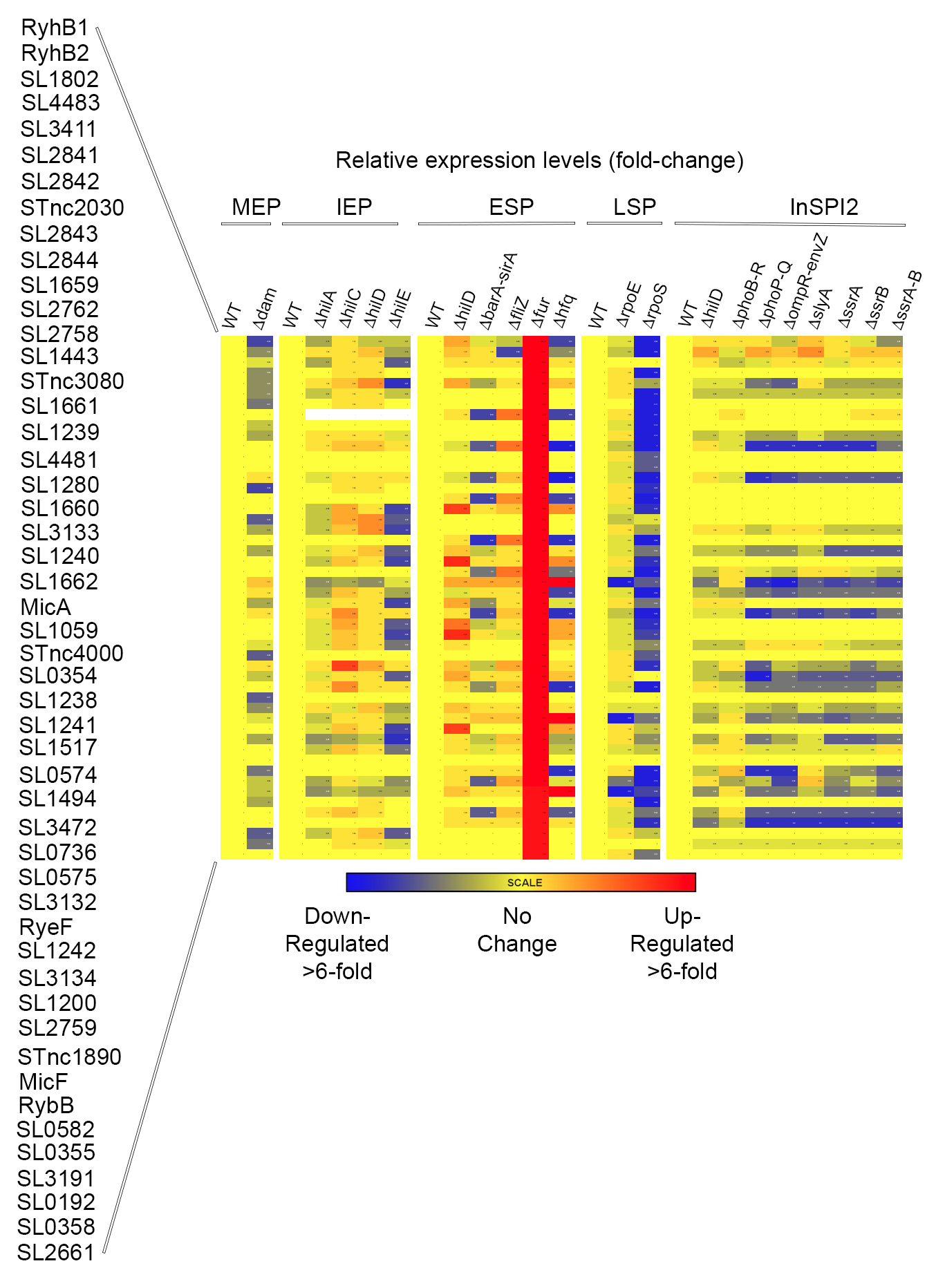

Supplement: S2 Fig — Heatmaps were recovered from the Salmonella SalCom database (http://bioinf.gen.tcd.ie/cgi-bin/salcom.pl?header_rotation=45;query=prpB;db=SalComRegulon_HL). As mentioned in Colgan et al. [8], “strains were grown either in Lennox broth to OD600 0.1 (EEP), 0.3 (MEP), 1.0 (LEP) 2.0 (ESP) and 2.0 + 6 h (LSP) or in the InSPI2 condition (slightly acidic pH and limitation of inorganic phosphate) which mimics aspects of the intra-macrophage conditions and induces expression of SPI2 Type 3 secretion system”. (TIF) [file pone.0265511.s002.tif]

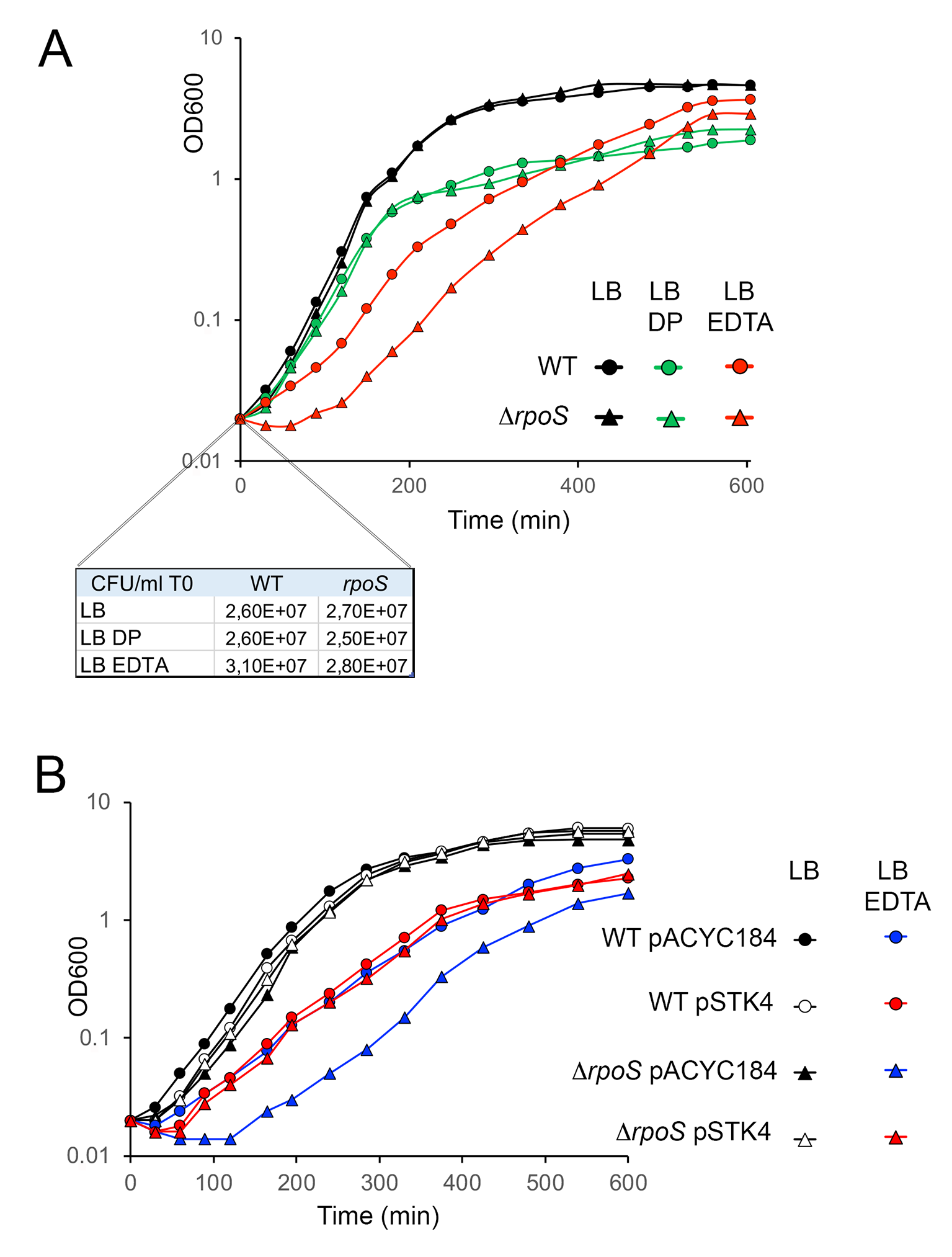

Supplement: S3 Fig — Biological replicates of that in Fig 7. (TIF) [file pone.0265511.s003.tif]

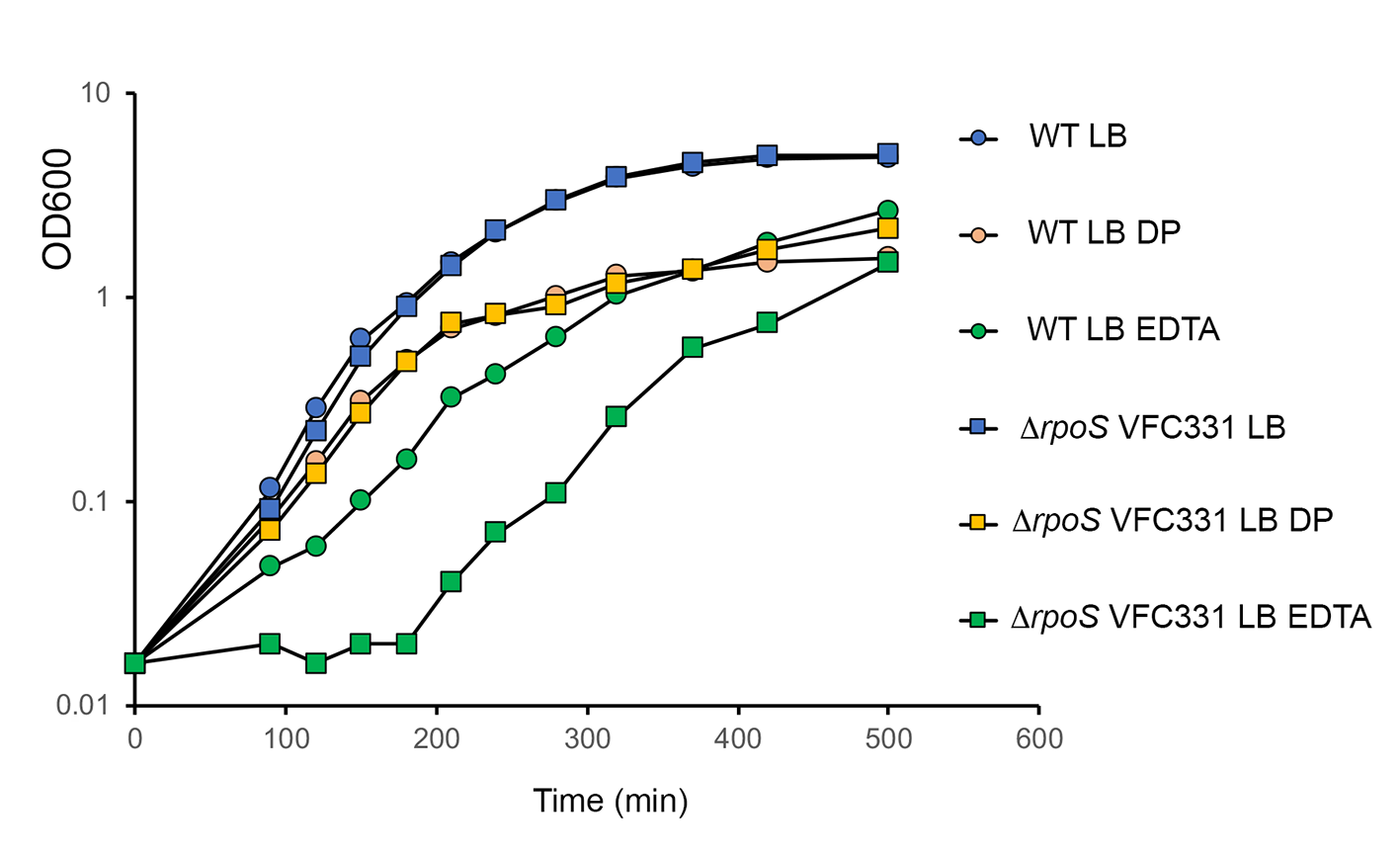

Supplement: S4 Fig — Same experiment as in Fig 7A–7D, but using the ΔrpoS mutant VFC331. (TIF) [file pone.0265511.s004.tif]

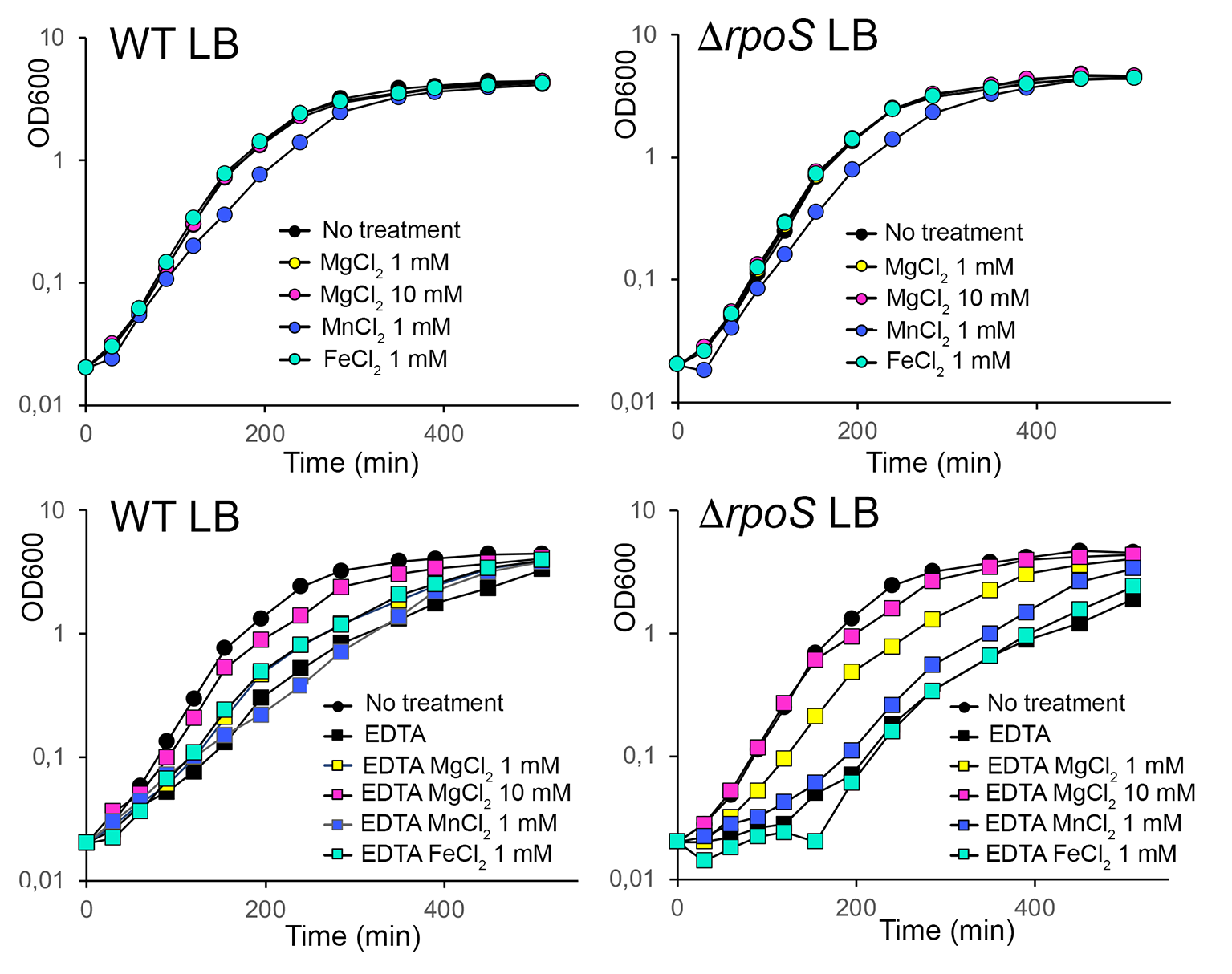

Supplement: S5 Fig — Independent repeat experiment of Fig 8. (TIF) [file pone.0265511.s005.tif]
