# Supplementary material for: The stress sigma factor σS/RpoS counteracts Fur repression of genes involved in iron and manganese metabolism and modulates the ionome of Salmonella enterica serovar Typhimurium
Source: PLoS One. 2022 Mar 31;17(3):e0265511. doi: 10.1371/journal.pone.0265511 (PMC8970401; doi:10.1371/journal.pone.0265511)
Supplement: S1 Table — (DOC) [file pone.0265511.s006.doc]

**S1 Table: Bacterial strains and plasmids used in this study**

| **Strain or Plasmid** | **Characteristics** | **Source or reference** |
| --- | --- | --- |
|  |  |  |
| VF6910 | ATCC14028 *Salmonella enterica* serovar Typhimurium, wild-type strain | American Type Culture Collection |
| VF8158 | ATCC14028 Δ*rpoS*ΔCm | (1) |
| VFC331 | ATCC14028 Δ*rpoS* (scarless in frame deletion of *rpoS*) | (2) |
| VF9480 | ATCC14028 Δ*fur-41::cat* | (3) |
| VF9523 | VF8158 Δ*fur-41::cat* | (3) |
| VFG582 | ATCC14028 Δ*mntR::*Km | This study |
| VFH380 | VF8158 Δ*mntR::*Km | This study |
| VFH382 | VF9523 Δ*mntR::*Km | This study |
| VFH400 | VFH380 with the Km cassette eliminated | This study |
| VFH402 | VFH382 with the Km cassette eliminated | This study |
| VFF546 | VF6910 *cbiO-lac*40 | This study |
| VFF547 | VF8158 *cbiO-lac*40 | This study |
| VFH130 | VF6910 *sufS-lac*40 | This study |
| VFH131 | VF8158 *sufS-lac*40 | This study |
| VFH132 | VF9480 *sufS-lac*40 | This study |
| VFH133 | VF9523 *sufS-lac*40 | This study |
| VFH122 | VF6910 STM14_5469-*lac*40 | This study |
| VFH123 | VF8158 STM14_5469-*lac*40 | This study |
| VFH124 | VF9480 STM14_5469-*lac*40 | This study |
| VFH125 | VF9523 STM14_5469-*lac*40 | This study |
| VFH343 | VF6910 *iroB-lac*36 | This study |
| VFH344 | VF8158 *iroB-lac*36 | This study |
| VFH345 | VF9480 *iroB-lac*36 | This study |
| VFH346 | VF9523 *iroB-lac*36 | This study |
| VFH347 | VF6910 *iroN-lac*36 | This study |
| VFH348 | VF8158 *iroN-lac*36 | This study |
| VFH349 | VF9480 *iroN-lac*36 | This study |
| VFH350 | VF9523 *iroN-lac*36 | This study |
| VFH367 | VF6910 *sitA-lac*36 | This study |
| VFH368 | VFC331 *sitA-lac*36 | This study |
| VFH369 | VF9480 *sitA-lac*36 | This study |
| VFH370 | VF9523 *sitA-lac*36 | This study |
| VFH434 | VFH400 *sitA-lac*36 | This study |
| VFH436 | VFH402 *sitA-lac*36 | This study |
| VFH363 | VF6910 *mntH-lac*36 | This study |
| VFH364 | VFC331 *mntH-lac*36 | This study |
| VFH365 | VF9480 *mntH-lac*36 | This study |
| VFH366 | VF9523 *mntH-lac*36 | This study |
| VFH431 | VFH400 *mntH-lac*36 | This study |
| VFH433 | VFH402 *mntH-lac*36 | This study |
| VFG300 | VF6910 *corA-lac*36 | This study |
| VFG301 | VF8158 *corA-lac*36 | This study |
| VFI24 | VF6910 *rcnA-lac*36 | This study |
| VFI25 | VF8158 *rcnA-lac*36 | This study |
| VFH947 | VF6910 *trkA-lac*36 | This study |
| VFH982 | VF8158 *trkA-lac*36 | This study |
| VFH949 | VF6910 *kdpA-lac*36 | This study |
| VFH983 | VF8158 *kdpA-lac*36 | This study |
| VFH951 | VF6910 *kefF-lac*36 | This study |
| VFH984 | VF8158 *kefF-lac*36 | This study |
| VFH953 | VF6910 *ybgR-lac*36 | This study |
| VFH985 | VF8158 *ybgR-lac*36 | This study |
| VFH941 | VF6910 *trkD-lac*36 | This study |
| VFH979 | VF8158 *trkD-lac*36 | This study |
| VFH943 | VF6910 *kefB-lac*36 | This study |
| VFH980 | VF8158 *kefB-lac*36 | This study |
| VFH945 | VF6910 *ycgO-lac*36 | This study |
| VFH981 | VF8158 *ycgO-lac*36 | This study |
| VFE259 | VF6910 *ryhB1-lac*36 | This study |
| VFE260 | VF8158 *ryhB1-lac*36 | This study |
| VFE261 | VF9480 *ryhB1-lac*36 | This study |
| VFE262 | VF9523 *ryhB1-lac*36 | This study |
| VFF85 | VF6910 *ryhB1-mut1-lac*36 | This study |
| VFF86 | VF8158 *ryhB1-mut1-lac*36 | This study |
| VFF87 | VF9480 *ryhB1-mut1-lac*36 | This study |
| VFF88 | VF9523 *ryhB1-mut1-lac*36 | This study |
| VFF89 | VF6910 *ryhB1-mut2-lac*36 | This study |
| VFF90 | VF8158 *ryhB1-mut2-lac*36 | This study |
| VFF91 | VF9480 *ryhB1-mut2-lac*36 | This study |
| VFF92 | VF9523 *ryhB1-mut2-lac*36 | This study |
| VFE263 | VF6910 *ryhB2-lac*36 | This study |
| VFE264 | VF8158 *ryhB2-lac*36 | This study |
| VFE265 | VF9480 *ryhB2-lac*36 | This study |
| VFE266 | VF9523 *ryhB2-lac*36 | This study |
| VFG190 | VF6910 *ryhB2-mut1-lac*36 | This study |
| VFG191 | VF8158 *ryhB2-mut1-lac*36 | This study |
| VFG192 | VF9480 *ryhB2-mut1-lac*36 | This study |
| VFG193 | VF9523 *ryhB2-mut1-lac*36 | This study |
| VFG194 | VF6910 *ryhB2-mut2-lac*36 | This study |
| VFG195 | VF8158 *ryhB2-mut2-lac*36 | This study |
| VFG196 | VF9480 *ryhB2-mut2-lac*36 | This study |
| VFG197 | VF9523 *ryhB2-mut2-lac*36 | This study |
| VFG198 | VF6910 *ryhB2-mut3-lac*36 | This study |
| VFG199 | VF8158 *ryhB2-mut3-lac*36 | This study |
| VFG200 | VF9480 *ryhB2-mut3-lac*36 | This study |
| VFG201 | VF9523 *ryhB2-mut3-lac*36 | This study |
|  |  |  |
| **Plasmids** |  |  |
| pACYC184 | Cloning vector, Cm^R^, Tet^R^ | (4) |
| pSTK4 | *rpoS* cloned into pACYC184, Cm^R^ | (5) |

1. Robbe-Saule V, Jaumouille V, Prevost MC, Guadagnini S, Talhouarne C, Mathout H, et al. Crl activates transcription initiation of RpoS-regulated genes involved in the multicellular behavior of Salmonella enterica serovar Typhimurium. Journal of bacteriology. 2006;188(11):3983-94.

2. Levi-Meyrueis C, Monteil V, Sismeiro O, Dillies MA, Monot M, Jagla B, et al. Expanding the RpoS/sigmaS-network by RNA sequencing and identification of sigmaS-controlled small RNAs in Salmonella. PloS one. 2014;9(5):e96918.

3. Monteil V, Kolb A, D'Alayer J, Beguin P, Norel F. Identification of conserved amino acid residues of the Salmonella sigmaS chaperone Crl involved in Crl-sigmaS interactions. Journal of bacteriology. 2010;192(4):1075-87.

4. Chang AC, Cohen SN. Construction and characterization of amplifiable multicopy DNA cloning vehicles derived from the P15A cryptic miniplasmid. Journal of bacteriology. 1978;134(3):1141-56.

5. Kowarz L, Coynault C, Robbe-Saule V, Norel F. The Salmonella typhimurium katF (rpoS) gene: cloning, nucleotide sequence, and regulation of spvR and spvABCD virulence plasmid genes. Journal of bacteriology. 1994;176(22):6852-60.
