# Supplementary material for: The stress sigma factor σS/RpoS counteracts Fur repression of genes involved in iron and manganese metabolism and modulates the ionome of Salmonella enterica serovar Typhimurium
Source: PLoS One. 2022 Mar 31;17(3):e0265511. doi: 10.1371/journal.pone.0265511 (PMC8970401; doi:10.1371/journal.pone.0265511)
Supplement: S2 Table — (DOC) [file pone.0265511.s007.doc]

**S2 Table.** **Oligonucleotides used in this study.**

| Name | Sequence (5’ – 3’) | Construction/Purpose |
| --- | --- | --- |
| cbiO-P4 | GTTTTTTCATCGAATGCAAAAGTGCGCATTCAGGGAGGCGTCAATTCCGGGGATCCGTCGACC | Translational *lacZ* fusion *cbiO-lac40* |
| cbiO-P1R | CCGACGTCGGATGCCGTCCCCTGCAACATAACTGCCTGCGTCAGTGTAGGCTGGAGCTGCTTC | Translational *lacZ* fusion *cbiO-lac40* |
| sufS-P4 | CCGACTGGTGGCAGGATTAACGCGTATCCACCGCTTATTGGGAATTCCGGGGATCCGTCGACC | Translational *lacZ* fusion *sufS-lac40* |
| sufS-P1R | GTTTTTCTTTATCCGGTAGCGCAGCCATAAAACCTCACTGTTAGTGTAGGCTGGAGCTGCTTC | Translational *lacZ* fusion *sufS-lac40* |
| iroB-P1 | TCTCATGCGTATTCTGTTTGTCGGTCCACCACTGTATGGACTGGTGTAGGCTGGAGCTGCTTC | Transcriptional *lacZ* fusion *iroB-lac36* |
| iroB-P2 | GTTCAGCCGCCATATTGTCATTGCGCTGCCGCGGTTAGCCGTGCATATGAATATCCTCCTTAG | Transcriptional *lacZ* fusion *iroB-lac36* |
| 5469-P4 | CATTATCAATTCCAGAGGTGAAACCATGTTGCAGCGGACGTTAATTCCGGGGATCCGTCGACC | Translational *lacZ* fusion STM14_5469-*lac40* |
| 5469-P1R | CAACGATAATCACTCCAGGCAATAATACGCCCCATCCGCTGCCGTGTAGGCTGGAGCTGCTTC | Translational *lacZ* fusion STM14_5469-*lac40* |
| iroN-P1 | GGGAATGGGTATGAGAGTTAAGAAGTTCATCTGGTTAATAACCGTGTAGGCTGGAGCTGCTTC | Transcriptional *lacZ* fusion *iroN-lac36* |
| iroN-P2 | CCAGAATATGCCCGCCAGCGGCGGGCATAATCCATCAAAACGACATATGAATATCCTCCTTAG | Transcriptional *lacZ* fusion *iroN-lac36* |
| sitA-P1 | TAACCACATTGTTGCGAGGGATACTATGACGAATCTACATCGTGTGTAGGCTGGAGCTGCTTC | Transcriptional *lacZ* fusion *sitA-lac36* |
| sitD-P2 | ATTATGACCGATTGTTTCCTGAATAGCAGGCAAAAAAGTGTTACATATGAATATCCTCCTTAG | Transcriptional *lacZ* fusion *sitA-lac36* |
| mntH-P1 | AGCAAAGGCTATGTTTTTGAGGCAAAAGATGACTGACAATCGCGTGTAGGCTGGAGCTGCTTC | Transcriptional *lacZ* fusion *mntH-lac36* |
| mntH-P2 | CCGATGACGCCCACGCATCGGGCCTGCTATCTTTCTGTCTTTACATATGAATATCCTCCTTAG | Transcriptional *lacZ* fusion *mntH-lac36* |
| corA-P1 | CGCATTGGGAGTCCCGGTCATGCTGAGCGCATTTCAACTGGAAGTGTAGGCTGGAGCTGCTTC | Transcriptional *lacZ* fusion *corA-lac36* |
| corA-P2 | CAGCCGCAGCTGAATCACCCTGGCCTTAATGTCTTACAGCCACATATGAATATCCTCCTTAG | Transcriptional *lacZ* fusion *corA-lac36* |
| rcnA-P1 | CATAAAACAACTAAGAATCATTCTCATGGGTGAATTTTCGACAGTGTAGGCTGGAGCTGCTTC | Transcriptional *lacZ* fusion *rcnA-lac36* |
| rcnA-P2 | ATGATACGGCCTGAGCAGAAGCGTTACATATTTACTGCATGATCATATGAATATCCTCCTTAG | Transcriptional *lacZ* fusion *rcnA-lac36* |
| trkA-P1 | GTGATGAGATAACGGGCCACGACTGATGAAAATTATCATACTGGTGTAGGCTGGAGCTGCTTC | Transcriptional *lacZ* fusion *trkA-lac36* |
| trkA-P2 | AAAGGGAATTAAAAAGGGCGCTGAATGAGCGCCCCTTAAATTACATATGAATATCCTCCTTAG | Transcriptional *lacZ* fusion *trkA-lac36* f |
| kdpA-P1 | CCCTGATTAATGCGGAGGCGTTCTGTAGGCCGCGCAAGGATTTGTGTAGGCTGGAGCTGCTTC | Transcriptional *lacZ* fusion *kdpA-lac36* |
| kdpE-P2 | CATAAAAATTAGAAGTTATTTAAAAATAATGAATAGCTATTCACATATGAATATCCTCCTTAG | Transcriptional *lacZ* fusion *kdpA-lac36* |
| kefF-P1 | AAAAACAGATCATCACAGGCTAATCATGATCCTCATTATTTATGTGTAGGCTGGAGCTGCTTC | Transcriptional *lacZ* fusion *kefF-lac36* |
| kefC-P2 | AAGTTTGCAGAACATCACATTTTTTACTTTAGATTGACGGTTTCATATGAATATCCTCCTTAG | Transcriptional *lacZ* fusion *kefF-lac36* |
| ybgR-P1 | ATGAGAATAACAAAGGAATGACGTTATGGCGCACTCACACTCTGTGTAGGCTGGAGCTGCTTC | Transcriptional *lacZ* fusion *ybgR-lac36* |
| ybgR-P2 | GGGAGCGAGGATCGCGCGCGCTTTCCCGTTAATGGTGATGAACCATATGAATATCCTCCTTAG | Transcriptional *lacZ* fusion *ybgR-lac36* |
| trkD-P1 | ACGTGTATAAACGAAAGATGAGTCTATGAGCACTGATAATAAGGTGTAGGCTGGAGCTGCTTC | Transcriptional *lacZ* fusion *trkD-lac36* |
| trkD-P2 | CCGGGTAAGCCAACGCGGCCCCCGGCATGTTGATGGGAGGTTACATATGAATATCCTCCTTAG | Transcriptional *lacZ* fusion *trkD-lac36* |
| kefB-P1 | ATCCTGTGAGCGCGGGAGGCTATTGATGGAAGGTGCTGATTTAGTGTAGGCTGGAGCTGCTTC | Transcriptional *lacZ* fusion *kefB-lac36* |
| kefB-P2 | GCCTGCAATAAAACGTTTACGGATTGCCATTGTTTTACCCCTACATATGAATATCCTCCTTAG | Transcriptional *lacZ* fusion *kefB-lac36* |
| ycgO-P1 | GTGAGTCAGGAGATATAAAACGTTGGATGCTGCAACAATTATTGTGTAGGCTGGAGCTGCTTC | Transcriptional *lacZ* fusion *ycgO-lac36* |
| ycgO-P2 | ACCGGTCCACTTGGGGGCGGGTCGACGTTGCCAGGCGACCTTACATATGAATATCCTCCTTAG | Transcriptional *lacZ* fusion *ycgO-lac36* |
| ryhB1-P1 | CTGCGAATGAGAATGATTATTATTGCCTTGCATTCAGGGGAACGTGTAGGCTGGAGCTGCTTC | Transcriptional *lacZ* fusion *ryhB1-lac36* |
| ryhB1-P2 | GAAGCAATGTGAGCAATGTCGTGCTTTCAGGTTCTCCGTAGGGCATATGAATATCCTCCTTAG | Transcriptional *lacZ* fusion *ryhB1-lac36* f |
| ryhB1-mut1-P1 | GAAGTAGACAACTGCGGCGGAGAATGATTATTATTGCCTTGCATTCAGGGGAACGTGTAGGCTGGAGCTGCTTC | Transcriptional *lacZ* fusion *ryhB1*_mut1_*-lac36* |
| ryhB1-P2 | GAAGCAATGTGAGCAATGTCGTGCTTTCAGGTTCTCCGTAGGGCATATGAATATCCTCCTTAG | Transcriptional *lacZ* fusion *ryhB1*_mut1_*-lac36* |
| ryhB1-mut2-P1 | GAAGTAGACAACTGCGAATGAGGCGGATTATTATTGCCTTGCATTCAGGGGAACGTGTAGGCTGGAGCTGCTTC | Transcriptional *lacZ* fusion *ryhB1*_mut2_*-lac36* |
| ryhB1-P2 | GAAGCAATGTGAGCAATGTCGTGCTTTCAGGTTCTCCGTAGGGCATATGAATATCCTCCTTAG | Transcriptional *lacZ* fusion *ryhB1*_mut2_*-lac36* |
| ryhB2-P1 | CATTAATGATAACGATTATCTTTATCAATACCGAGTGGTTGAGTGTAGGCTGGAGCTGCTTC | Transcriptional *lacZ* fusion *ryhB2-lac36* |
| ryhB2-P2 | AGCAATGTGAGCAATGTCGTACCGAACAGGTGGGTTATAAACCATATGAATATCCTCCTTAG | Transcriptional *lacZ* fusion *ryhB2-lac36* |
| ryhB2-mut1-P1 | TATCAGGGTTGCACGCATTAATGATAACGATTATCTTTATCAATACCGAGTGGTTGAGTGTAGGCTGGAGCTGCTTC | Transcriptional *lacZ* fusion *ryhB2*_mut1_*-lac36* |
| ryhB2-P2 | AGCAATGTGAGCAATGTCGTACCGAACAGGTGGGTTATAAACCATATGAATATCCTCCTTAG | Transcriptional *lacZ* fusion *ryhB2*_mut1_*-lac36* |
| ryhB2-mut2-P1 | TATCAGGGTTGCAAGCATTAATGATAACGATTATCTTTATCAATACCGAGTGGTTGAGTGTAGGCTGGAGCTGCTTC | Transcriptional *lacZ* fusion *ryhB2*_mut2_*-lac36* |
| ryhB2-P2 | AGCAATGTGAGCAATGTCGTACCGAACAGGTGGGTTATAAACCATATGAATATCCTCCTTAG | Transcriptional *lacZ* fusion *ryhB2*_mut2_*-lac36* |
| ryhB2-mut3-P1 | CAGGGTTGCAATCATTGCGGATAACGATTATCTTTATCTTTATCAATACCGAGTGGTTGAGTGTAGGCTGGAGCTGCTTC | Transcriptional *lacZ* fusion *ryhB2*_mut3_*-lac36* |
| ryhB2-P2 | AGCAATGTGAGCAATGTCGTACCGAACAGGTGGGTTATAAACCATATGAATATCCTCCTTAG | Transcriptional *lacZ* fusion *ryhB2*_mut3_*-lac36* |
| mntR-P1 | CGCGTCACAGAAACGAGGAAGCAAAATGGGTCGTCGCGCAGGTGTGTAGGCTGGAGCTGCTTC | Δ*mntR*::Km |
| mntR-P2 | AAAAGGCCAGACTCATTCTGCAGATGTTCCATGCTGTTGCGTCATATGAATATCCTCCTTAG | Δ*mntR*::Km |
| FNB-RyhB1 | GTGCTTTCAGGTTCTCCGTAGGGGTTCCC | Antisens oligo to probe for RyhB1 sRNA (1) |
| FNB-RyhB2 | ACCGAACAGGTGGGTTATAAACTCAACCACTCGGT | Antisens oligo to probe for RyhB2 sRNA (1) |
| CL-5S | GAGACCCCACACTACCATC | Antisens oligo to probe for 5S sRNA (1) |

1. Levi-Meyrueis C, Monteil V, Sismeiro O, Dillies MA, Monot M, Jagla B, et al. Expanding the RpoS/sigmaS-network by RNA sequencing and identification of sigmaS-controlled small RNAs in Salmonella. PloS one. 2014;9(5):e96918.
