# Supplementary material for: The stress sigma factor σS/RpoS counteracts Fur repression of genes involved in iron and manganese metabolism and modulates the ionome of Salmonella enterica serovar Typhimurium
Source: PLoS One. 2022 Mar 31;17(3):e0265511. doi: 10.1371/journal.pone.0265511 (PMC8970401; doi:10.1371/journal.pone.0265511)

Fig 1 Panels ABCEFG

(AB and EF) WT (1) Δ*rpoS* (2) Δ*fur* (3) and Δ*fur*Δ*rpoS* (4) strains grown in LB

(CG) WT strain grown in LB (5), LB+ DP (6), LB + $FeCl_2$ (7)

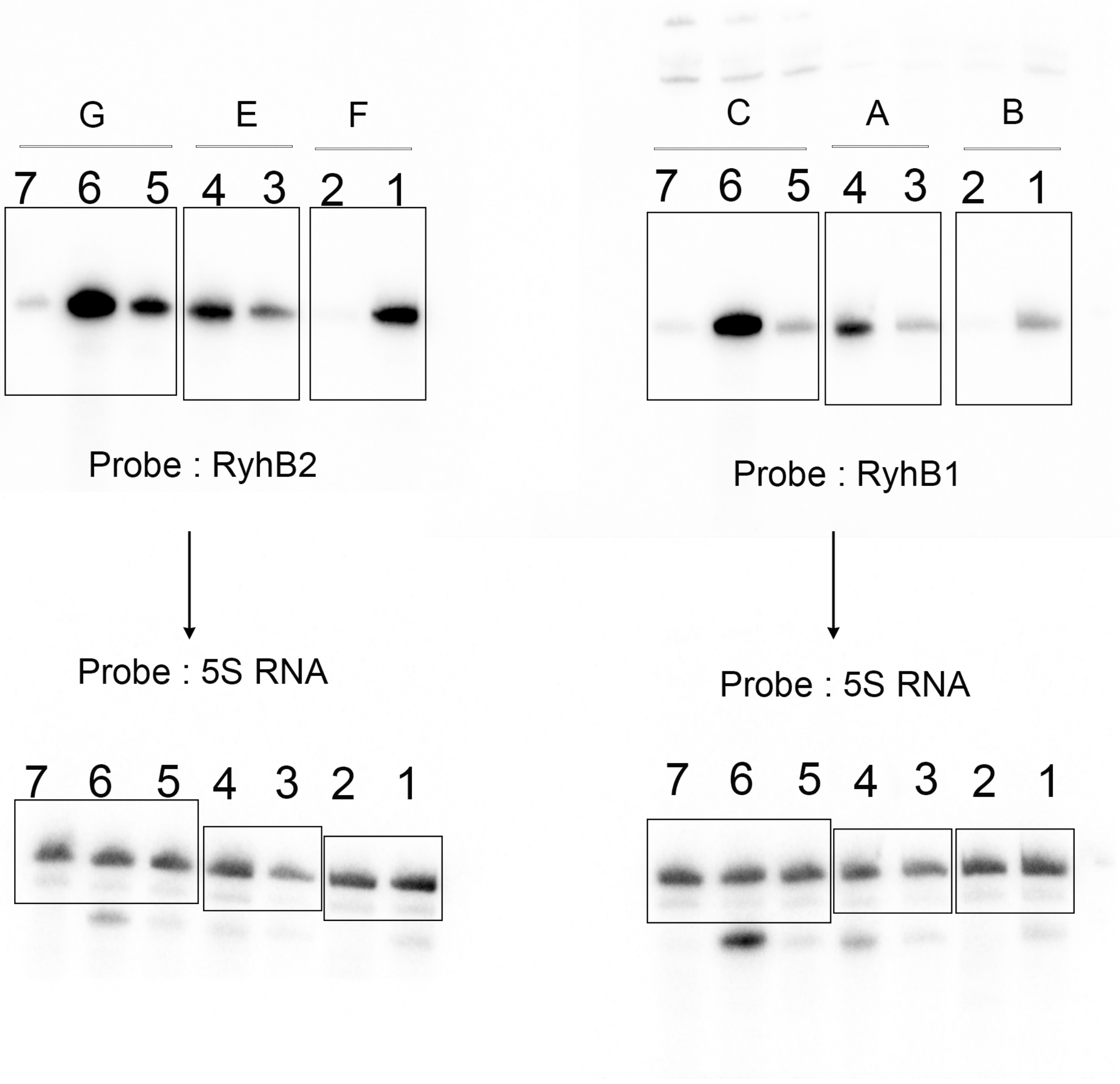

Fig 1 Panels D and H
RyhB1 and RyhB2 sRNAs

Δ*rpoS* mutant grown in
LB (1), LB+ DP (2), LB + $FeCl_2$ (3)

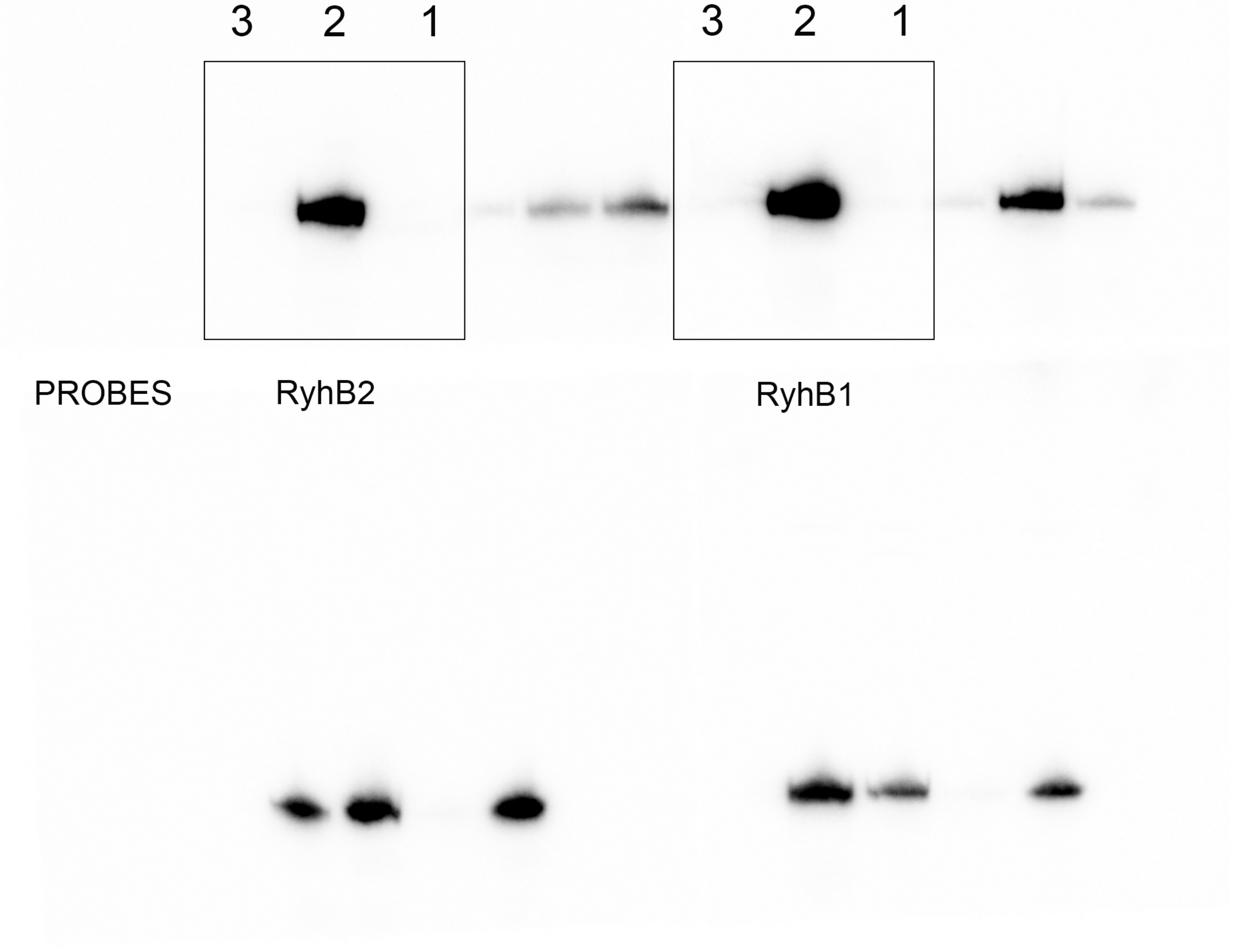

Fig 1 Panels D and H
5S RNA

Δ*rpoS* mutant grown in
LB (1), LB+ DP (2), LB + $FeCl_2$ (3)

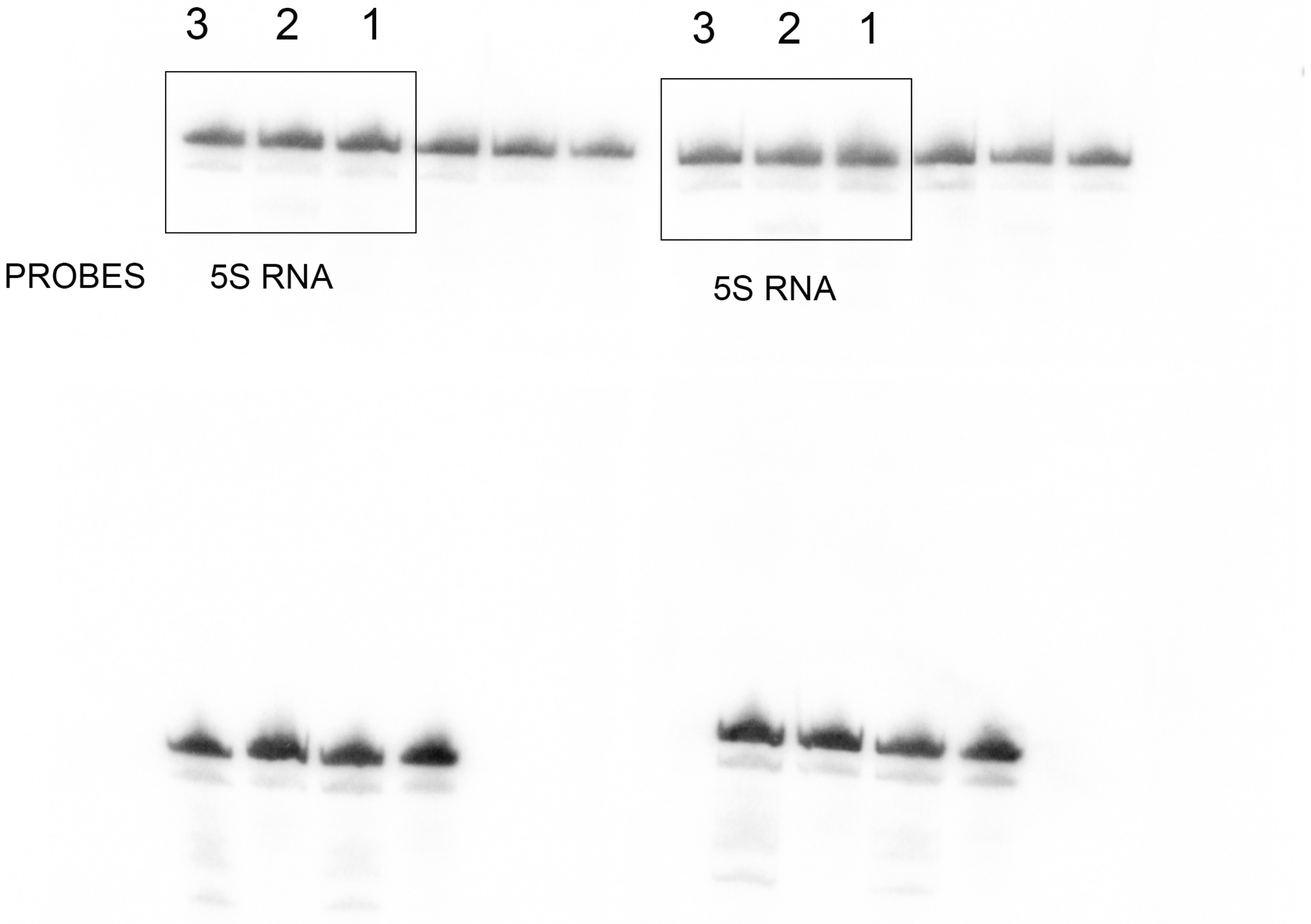

Supplement: S1 Raw images — (PDF) [file pone.0265511.s009.pdf]
